# Plasma cells, plasmablasts, and AID^+^/CD30^+^ B lymphoblasts inside and outside germinal centres: details of the basal light zone and the outer zone in human palatine tonsils

**DOI:** 10.1007/s00418-020-01861-1

**Published:** 2020-03-14

**Authors:** Birte S. Steiniger, Linda Raimer, Anja Ecke, Boris A. Stuck, Yalcin Cetin

**Affiliations:** 1 Institute of Anatomy and Cell Biology, University of Marburg, Robert-Koch-Str. 8, 35037 Marburg, Germany; 2 Department of Otorhinolaryngology, University Hospital Marburg, University of Marburg, Marburg, Germany

**Keywords:** Palatine tonsils, Germinal centres, Plasma cells, Plasmablasts, B lymphoblasts

## Abstract

Plasma cells (PCs) in human palatine tonsils are predominantly located in the germinal centres (GCs), in the subepithelial space and near the deep connective tissue septa surrounding each crypt. We analysed the location, phenotype, and proliferation of GC PCs by immunohistology comparing them to PCs in the other two locations. Most PCs in GCs were strongly positive for CD38, CD138, CD27, IRF4, and intracellular (ic) IgG. They often accumulated in the basal light zone, but could also be found scattered in the entire light zone. In addition, rows of PCs occurred at the surface of the GC bordering the mantle zone, i.e., in the outer zone, and at the surface of the dark zone. The latter cells were often continuous with PCs in the extrafollicular area. The vast majority of GC PCs were negative for Ki-67. Only a few Ki-67^+^ plasmablasts, predominantly icIgG^+^ or icIgM^+^, were found inside GCs. In certain GCs PCs accumulated around capillaries and the adjacent perikarya of follicular dendritic cells (FDCs). Newly formed PCs might migrate from the basal to the superficial part of the light zone and then back to the dark zone surface to leave the GC. This guarantees an even distribution of secreted Ig for exchange with immune complexes on FDCs. The surface of the dark zone may also be an exit site for Ki-67^+^CD30^+^ B lymphoblasts, which seed perifollicular and extrafollicular sites. We speculate that these cells tend to downmodulate CD20 and activation-induced deaminase and further up-regulate CD30 when developing into pre-plasmablasts.

## Introduction

PCs have been known as regular and potentially abundant constituents of secondary follicles in human tonsils and in the lymphatic organs of experimental animals for about 50 years (Lennert et al. 1967; Curran and Jones 1977; Korsrud and Brandtzaeg 1980; Nieuwenhuis and Opstelten 1984; Arpin et al. 1998). In spite of this, most models of GC function published up to now have ignored this fact (De Silva and Klein 2015; Mesin et al. 2016; Habermann et al. 2019). GC models are predominantly based on findings during short-term B-cell reactions to hapten antigens in mouse lymph nodes or spleen which seldom exceed 4 weeks. Current models assume that only the initial proliferation-associated steps of PC differentiation, i.e., plasmablasts, occur inside GCs. Final PC differentiation is assumed not to start before plasmablasts have left GCs to distribute either locally and/or in the entire body via lymphatics and the bloodstream (Zhang et al 2018; Mohr et al. 2009).

It is likely that GCs in humans and especially in tonsils are, however, long-lasting. Tonsils are permanently confronted with materials of viral, bacterial, and fungal origin in the oral and nasal cavity. Their GCs are most probably established by a large number of different B-cell clones responding to various antigenic molecules and to different epitopes in single antigens (Mesin et al. 2016; Küppers et al. 1993). In addition, tonsils are only removed if long-term—perhaps even overshooting—B-cell reactions provoke recurrent inflammation or spatial obstruction. Thus, tonsils are ideal for observing human B cells and plasma cells during long-lasting immune responses of high intensity. Tonsils do not occur in mice or rats, which means that immunological control of the upper aerodigestive tract is accomplished differently from humans. This may be related to the existence of bronchus-associated lymphatic tissue (Randall 2010) and to a relatively large number of Peyer’s patches in the gut wall in both species.

The overall structure of human tonsil GCs has been described previously without special reference to PCs (Hardie et al. 1993; Brachtel et al. 1996). We adopt the terminology used in these publications, namely that human tonsil GCs are surrounded by a mantle zone and consist of a dark zone, a basal and an apical light zone and an outer zone. The latter zone is ill-defined and was originally characterised by the absence of CD23^+^ FDCs (Brachtel et al. 1996). In a subsequent study, it became, however, clear that not all tonsil GCs had such a CD23^−^ area (Steiniger et al. 2011). Thus, the microanatomy of tonsil GCs does not necessarily differ from that of lymph-node GCs as supposed by Brachtel et al. (1996).

The existing evidence for plasma cells inside tonsil GCs has not answered the question whether cells exhibiting large amounts of icIg in this location have to be classified as proliferating plasmablasts or as non-proliferating plasma cells. The most widely accepted feature discriminating both cell types is the expression of Ki-67, especially in immunohistological investigations, although proliferation may not be the only criterion for differentiating human plasmablasts from plasma cells.

In humans, among many other antigens, CD27, CD38, and CD138 (Khodadadi et al. 2019; Mei et al. 2007), accumulation of icIg and nuclear staining for IRF4 (Klein et al. 2006) have all been shown to occur in plasmablasts and PCs. With exception of icIg, these molecules are not PC-specific and, thus, have to be investigated in double-staining experiments after careful analysis of their tissue distribution. Especially, CD27^+^ and CD38^+^ cells have to be distinguished from T cells. Human CD27^+^, CD38^+^, and CD138^+^ antibody-secreting cells have been demonstrated to increase in the blood after vaccination (Odendahl et al. 2005). Typically, it is shown that most of these cells express Ki-67 (Garimilla et al. 2019). A small number of CD38^+^Ki-67^−^ blood cells also exists after immunisation or in the steady state, but this cell type has not been analysed in detail up to now (Garimilla et al. 2019).

We detected cells with strong expression of CD38, CD27, CD138, and IRF4 and varying numbers of cells with icIgM, IgD, IgG, and IgA in single- and double-staining assays in the GCs of human palatine tonsils derived from 6 individuals of different ages. In addition, such cells were also found in spleen and lymph-node GCs. Cells with this phenotype exhibited a typical distribution pattern in secondary follicles. The vast majority of the cells were Ki-67^−^ and were classified as plasma cells. Only a few Ki-67^+^ plasmablasts with icIgM, IgD, IgG, or IgA were detected in human tonsil GCs. The majority of the PCs in GCs were CD38^++^, but a small population of CD38^−(or ±)^ PCs was also detected in double-staining experiments for CD38 and icIg.

Very few studies in mice have investigated which role soluble immunoglobulins (Igs) play for affinity maturation of differentiating B cells in GCs (Zhang et al. 2013, 2016, 2018). Although immunohistological demonstration of major amounts of intracellular Ig in PCs does not necessarily indicate Ig secretion, it is attractive to speculate that the immune complexes on follicular dendritic cells might be locally produced and modified by intra-GC PCs in long-lasting immune reactions.

## Materials and methods

### Specimens

We investigated 6 palatine tonsils obtained from male and female patients 3–57 years of age. Permission of the ethics committee of the Medical Faculty of Marburg University was obtained (No. 61/16). Within 1–2 h after removal, the tonsil specimens were fixed in 3.7% formaldehyde/tap water for 24 h. After washing in tap water, the specimens were transferred to and stored in 70% isopropanol via 50% isopropanol. Subsequently, the specimens were dehydrated and embedded in paraffin. The staining results in tonsils were always compared to those in a standard spleen specimen from a 22-year-old healthy individual processed by the same method. All primary antibodies directed to cell membrane antigens had been pre-tested for reactivity in tonsil cryosections of different individuals. One of the specimens, derived from a 57-year-old male individual, was used to produce 300 serial paraffin sections for triple staining of smooth muscle alpha-actin (SMA), CD38, and CD271.

A hyperplastic cervical lymph node from a 58-year-old male, a mesenteric lymph node (patient data unknown) removed because of rectal carcinoma and six spleens from healthy adults were also investigated for CD38 (lymph nodes) or for CD38 and for CD3/CD27 (spleens) and compared to tonsils. These specimens had been kindly donated by Dr. P.J. Barth, Institute of Pathology of Marburg University Hospital before 2004. According to the regulations of the Medical Faculty of Marburg University, an ethics vote was not required at that time.

### Immunohistology

#### Single staining

The paraffin sections were de-waxed, autoclaved at 120 °C for 20 min, and treated with glucose oxidase (GO, Sigma-Aldrich Chemie GmbH, Munich, Germany, No. G-6641) at 100 U/ml in PBS, pH 7.2, containing 20 mM beta-d-glucose and 2 mM NaN_3_) for 1 h at 37° to remove endogenous peroxidase. The antibodies listed in Table 1 were diluted in PBS/1% BSA/0.1% NaN3 plus 0.003 mg/ml avidin and incubated overnight. The Vectastain ABC elite Kit (Vector Laboratories, Burlingame, CA, USA, No. PK-6100 via Linaris, Wertheim, Germany) was used for detecting primary antibody binding with 0.02 mg/ml biotin contained in the biotinylated secondary anti-mouse (Vector Labs, No. BA-9200 via Linaris) or anti-rabbit (Vector Labs, No. BA-1000, via Linaris) antibody. Avidin and biotin were both added to inhibit potential endogenous avidin-binding activities in the sections by a very low concentration of free avidin and then saturated by biotin. Diaminobenzidine (DAB) was used as chromogen, and the sections were dehydrated and coverslipped in Eukitt. With exception of IgG, intracellular staining for Igs was achieved using polyclonal antibodies (Table 1) at high dilutions, so that cell surface staining was strongly reduced or absent. MAb 137B4 (anti-CD27) was routinely used with the ABC/DAB system and tyramide amplification as described (Steiniger et al. 2005). This staining method was also applied if increased staining intensity of anti-CD30 mAb Ber-H2 was desirable. For detection of AID (activation-induced deaminase) with mAb ZA001, the AP-based system mentioned below was more appropriate than ABC/DAB with or without tyramide amplification.

**Table 1:** Antibodies used

MAb clone name	Antigen	Distributor (Germany)
1B12	CD23	Bio-Rad/Serotec, Puchheim, No. MCA 1810 T
137B4	CD27	Quartett, Berlin, No. 030410901
B-B4	CD138	Bio-Rad/Serotec No. MCA 681H via Biozol, Eching
Ber-H2	CD30	Agilent/DAKO, Hamburg, No. M0751
EK25G9	AID	kind gift of G. Niedobitek, Inst. of Pathology, University of Erlangen, Germany
EP190	IRF4	Cell Marque via Medac, Wedel, No. 358R-14
EP1039Y	CD271	GeneTex via Biozol, Eching No. GTX61425
KiM1-P	CD68	gift of R. Parwaresch, Inst. of Pathology, University of Kiel, Germany
L26	CD20	DAKO, Hamburg, No. M0755
MIB-5	Ki-67	gift of J. Gerdes, Research Center Borstel, Germany
QBend-10	CD34	Dianova, Hamburg, Germany, No. DLN-09135
RWP49	IgG	Novocastra via Leica Biosystems, Nussloch, No. NCL-L-IgG
SPC32	CD38	Novocastra via Leica Biosystems, Nussloch, No. NCL-CD38-290
ZA001	AID	Thermo Fisher Scientific, Schwerte, No. 39-2500
Polyclonal	IgM,	Agilent/DAKO, Hamburg, No. A 425
"	IgD	Agilent/DAKO, Hamburg, No. A 0093
"	IgE	Acris Antibodies, Herford, Germany, No. DP022
"	IgA	Agilent/DAKO, Hamburg, No. A 0262
"	SMA	Sino Biological via Biozol, Eching, No. 100125-T40
"	CD3, epsilon chain	Agilent/DAKO, Hamburg, No. A 0452

#### Double staining

Double staining for transmitted light microscopy is principally a subtractive procedure, i.e., cells stained with one chromogen cannot be stained with a second one. Only if a part of a cell is lightly stained or unstained in the first step, the second chromogen can be polymerised. Thus, co-expression of two antigens in a cell is only reliably revealed if the antigens occupy different locations.

The antibodies mentioned in Table 1 were also used in the double-staining experiments listed in Table 2. For detecting, whether plasma cells were proliferating, mAb MIB-5 was applied first and the other antibodies were used in the second step. The reverse combination was also tested, especially in the case of IgD. 137B4 could only be used after first staining CD3, because all human T cells strongly express CD27. For this reason, we only established that CD27^++^ GC cells were non-proliferating non-T cells, but did not investigate further antigens together with CD27.

**Table 2:** Double-staining experiments

First Ag (Ab)	Second Ag (Ab)
CD3 (polyclonal)	CD38 (SPC32)
CD3 (polyclonal)	CD27 (137B4)
CD3 (polyclonal)	CD30 (Ber-H2)
CD20 (L26)^a^	CD3 (polyclonal)
CD20 (L26)	CD271 (EP1039Y)
CD20 (L26)	CD38 (SPC32)
CD20 (L26)^a^	AID (ZA001)
CD20 (L26)^a^	CD30 (Ber-H2)
CD38 (SPC32)	IgM (polyclonal)
CD38 (SPC32)	IgD (polyclonal)
CD38 (SPC32)	IgG (RWP49)
CD38 (SPC32)	IgA (polyclonal)
CD38 (SPC32)	CD271 (EP1039Y)
CD38 (SPC32)	CD27 (137B4)
CD38 (SPC32)^a^	CD23 (1B12)
Ki-67 (MIB-5)	IgM (polyclonal)
Ki-67 (MIB-5)	IgD (polyclonal)
Ki-67 (MIB-5)	IgG (RWP49)
Ki-67 (MIB-5)	IgA (polyclonal)
Ki-67 (MIB-5)	CD38 (SPC32)
Ki-67 (MIB-5)	CD138 (BB-4)
Ki-67 (MIB-5)	CD30 (Ber-H2)
Ki-67 (MIB-5)^a^	AID (ZA001)
Ki-67 (MIB-5) + CD3	CD27 (137B4)
IgM (polyclonal)^a^	Ki-67 (MIB-5)
IgD (polyclonal)^a^	Ki-67 (MIB-5)
IgG (RWP49)^a^	Ki-67 (MIB-5)
IgA (polyclonal)^a^	Ki-67 (MIB-5)
IgG (RWP49)	IgM (polyclonal)
CD23 (1B12)	CD38 (SPC32)
CD138 (BB-4)	CD38 (SPC32)
IRF4 (EP190)	CD3 (polyclonal)
IRF4 (EP190)	CD38 (SPC32)
IRF4 (EP190)	CD30 (Ber-H2)
CD30 (Ber-H2)^a^	AID (ZA001)
CD271 (EP1039Y)	CD20 (L26)
CD38 (SPC32)^b^	CD20 (L26)
AID (ZA001)^a^	CD3 (polyclonal)
AID (ZA001)^a^	CD38 (SPC32)
CD68 (KiM1-P)^a^	AID (ZA001)
CD68 (KiM1-P)^a^	CD38 (SPC32)

All staining combinations not marked by superscript letters were detected with BrightVision/HighDef Blue AP or ABC/HighDef Blue Peroxidase in the first step and ABC/DAB in the second step. Ber-H2 was detected by ABC/DAB with and without tyramide amplification

^a^First primary antibody detected by ABC/DAB and second primary antibody by BrightVision/HighDef Blue AP or ABC/HighDef Blue Perox

^b^First primary antibody detected by ABC/DAB and second primary antibody by ABC/HistoGreen

In most, but not all, double-staining experiments, the first antigen was revealed in blue–black colour using the BrightVision AP polymer system for mouse IgG (ImmunoLogic, Amsterdam, The Netherlands via VWR, Darmstadt, Germany, No. VWRKDPVM55AP) or rabbit IgG (same distributor, No. VWRKDPVR55AP) and Enzo HighDef Blue for AP (Enzo Life Sciences, Lörrach, Germany, No. ADI-950-150) as chromogen. The second antigen was demonstrated by the ABC method with DAB. Initially, the Vector ABC Elite system with Enzo HighDef Blue for peroxidase (Enzo Life Sciences, Lörrach, Germany, No. ADI-950-151) was applied in the first step, but this chromogen was subsequently abandoned. HighDef Blue for peroxidase in combination with the ABC system yielded more amplification than Enzo High Def Blue for AP with BrightVision. In addition, the staining intensity of HighDef Blue for peroxidase could be further amplified with tyramide. However, the polymerisation product of the blue peroxidase chromogen was not stable for more than a few weeks, if used in double-staining experiments with DAB and mounted in a hydrophilic medium. Air-drying the stained sections and using an acrylate-based mounting medium (Eukitt, Sigma-Aldrich, No. 03989) did not prevent the decay of the chromogen.

All double-stained sections were coverslipped in Mowiol (Sigma-Aldrich, No. 324590). The blue–black colour of Enzo HighDef Blue AP was preserved for more than 6 months. In some experiments, ABC/DAB was used to reveal the first antigen and ABC followed by the peroxidase chromogen HistoGreen (Linaris, Wertheim, Germany, No. E109M) was applied for the second. In this case, the sections could be coverslipped in Eukitt.

Controls omitting either the first or the second primary antibody were included in each test.

#### Triple staining

For successive detection of smooth muscle alpha-actin (SMA), CD38, and CD271, a triple staining method was developed. Dewaxed paraffin sections were autoclaved and treated with glucose oxidase as described for single staining. Polyclonal anti-SMA (final dilution 1:20.000) and SPC32 (final dilution 1:1000) were mixed in PBS/BSA/NaN3 plus 0.003 mg/ml avidin and incubated overnight. Subsequently, SMA was detected using the ABC Elite Kit (rabbit) as described by the manufacturer plus 0.02 mg/ml biotin in the second antibody solution and DAB as chromogen followed by BrightVision anti-mouse Ig and Enzo HighDef Blue AP. Then, EP1039Y was applied overnight at 1:1500 and detected by BrightVision anti-rabbit Ig and PermaRed AP (Diagnostic BioSystems, Pleasanton, CA, USA via Zytomed Systems, Berlin, Germany, No. K049-AUTO). After the last chromogen incubation, the sections were coverslipped in Mowiol.

### Image acquisition

The microphotographs were acquired with a Zeiss Axiophot microscope equipped with a Canon EOS 60D camera (18 MP) and Canon 60D utility, version 2. The front lenses used were Zeiss Planapo 10×/0.32, Planapo 20×/0.60, Planapo 40×/1.0 Oil Iris, Planapo 63×/1.40 Oil with or without 1,25× or 1,6× Axiophot internal optovar lenses.

## Results

### Microanatomy of human palatine tonsils

**Fig. 1:**
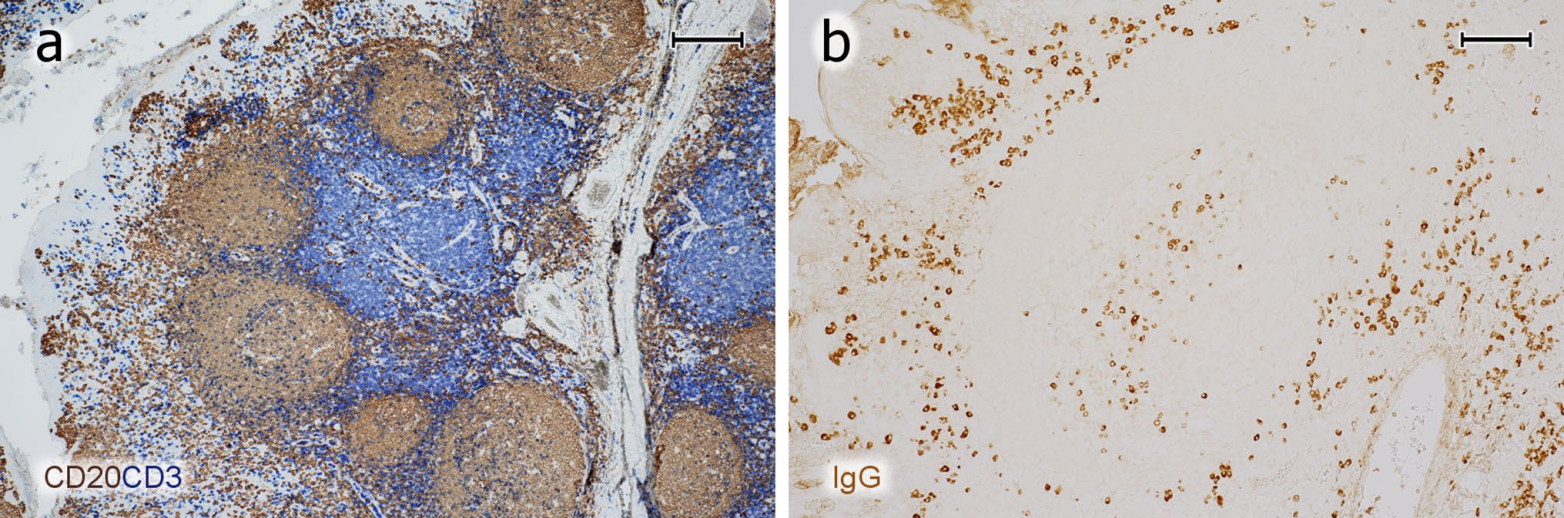
Overview of T- and B-cell regions and distribution of PCs in a representative tonsil. **a** Staining for CD20 (mAb L26) in brown followed by visualisation of CD3 (polyclonal Abs) in blue colour. A crypt is located to the left. The connective tissue septum (unstained) in the right part of the image separates the crypt and adjacent lymphatic tissue from the lymphatic tissue associated with the next crypt to the right. The follicles are surrounded by a small perifollicular region with mixed T and B cells. The T-cell zone proper (blue region) is located deeper in the lamina propria in proximity to the connective tissue septum. **b** IcIgG^+^ PCs are primarily located beneath the crypt epithelium (left), in a GC (center), and near the connective septum between two crypts containing a vein (right). **a**, **b** 38-year-old female. Scale bar 200 µm (**a**, **b**)

The overall structure of tonsils was first visualised by double-staining B and T lymphocytes for CD20 and CD3, respectively (Fig. 1a). Then, the distribution of icIgG^+^ or icIgM^+^ PCs was shown (Fig. 1b). The specimens contained parts of the tonsillar capsule giving rise to connective tissue septa in the organ. These septa were surrounded by the lamina propria and epithelium of one or several crypts. The epithelium near the ends of crypts and the adjacent lamina propria were heavily infiltrated by all types of lymphocytes and many plasma cells. In some tonsils the lamina propria formed papillary protrusions into the infiltrated epithelium. The lamina propria always contained secondary follicles with GCs and mantle zones. Perifollicular areas of mixed B- and T-cell composition surrounded the mantle zones. T-cell predominant regions often occurred deeper in the lamina propria located more closely to the connective tissue septum supporting the lamina propria between the crypts (Fig. 1a). This septum contained many small lymphatic vessels with podoplanin-positive lymph endothelium (not shown) and larger blood vessels.

When the six tonsil specimens investigated in the present study were compared to six tonsil specimens examined about 15 years ago (data not shown), it became clear that the present specimens exhibited secondary follicles of intermediate size. The follicles and GCs of the older specimens were much larger and they contained many more PCs, which may be due to different indications for tonsillectomy, to a change of therapies during recent decades, or to an unknown selection bias. In the present study, the size of follicles and GCs varied among different individuals irrespective of age. Care was taken to investigate organs of a broad age distribution.

### Visualisation of follicles

**Fig. 2:**
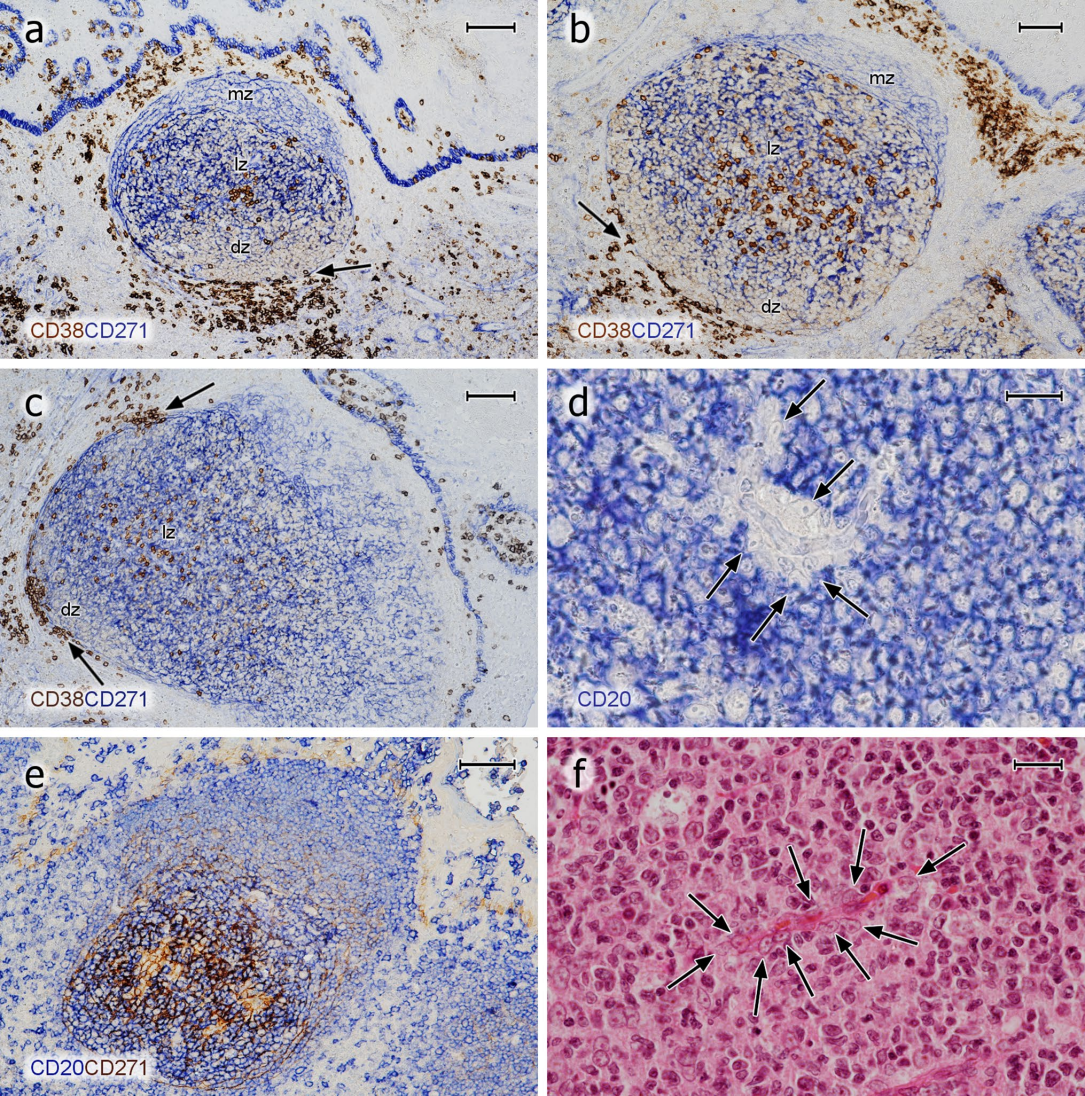
Distribution of CD38^++^ PCs and pericapillary FDCs in tonsil GCs. **a**–**c** Staining for CD38^++^ PCs (mAb SPC32, brown) followed by visualisation of CD271^+^ FDCs (mAb EP1039Y, blue). PCs may accumulate in the basal light zone and/or distribute evenly in the light zone and occur at the surface of the GC. They also form rows of cells at the dark zone surface, which are connected to PC clusters in the lamina propria outside the GC (arrows). The basal layer of the crypt epithelium is also CD271^+^. **d** FDCs at the surface of intra-GC microvessels (arrows) prevent CD20^+^ B lymphocytes (mAb L26, blue) from approaching the endothelium. **e** The unstained cells in **d** are CD271^+^, when CD20 is demonstrated in blue (mAb L26) followed by CD271 in brown (mAb EP1039Y). **f** The nuclei of the pericapillary FDCs (arrows) are also visible in routine HE-stained sections. *mz* mantle zone, *lz* light zone of GC, *dz* dark zone of GC. **a**, **b** 21-year-old female. **c** 12-year-old female. **d**, **e** 38-year-old female. **f** 57-year-old male. Scale bars 100 µm (**a**, **c**), 70 µm (**b**, **e**), 20 µm (**d**, **f**)

In previous publications, we (Steiniger et al. 2011, 2014a, b, 2018) and others (Maeda et al. 2002; Pezzati et al. 1992; Thompson et al. 1989) investigated the distribution of CD271 in tonsils and spleens. As reported, CD271 proved an optimal antigen for visualising follicles, because the staining precisely outlined FDCs and their processes in GCs, which alleviated double-staining experiments. FDCs in mantle zones were less intensely stained and formed a network with larger meshes compared to that in GCs. The GCs were distinguished from the mantle zones by a more intense expression of CD271 (Fig. 2a–c). In some follicles and individuals, however, FDCs in GCs and in mantle zones did not differ in staining intensity. Faint CD271 reactivity was also observed in the basal layer of the crypt epithelium and in interfollicular fibroblasts (Fig. 2a–c). When CD20^+^ B cells were demonstrated in follicles, an unstained space was often observed surrounding capillaries in GCs. In this space, cells with elongated or triangular nuclei and prominent nucleoli were present, which appeared to be FDCs positive for CD271 (Fig. 2d, e) and negative for CD68 (not shown). The nuclei and bodies of these cells were also visible in HE-stained sections (Fig. 2f). They seemed to prevent the CD20^+^ B cells from contacting the capillary endothelium.

### Distribution and preliminary phenotype of PCs in tonsils

**Fig. 3:**
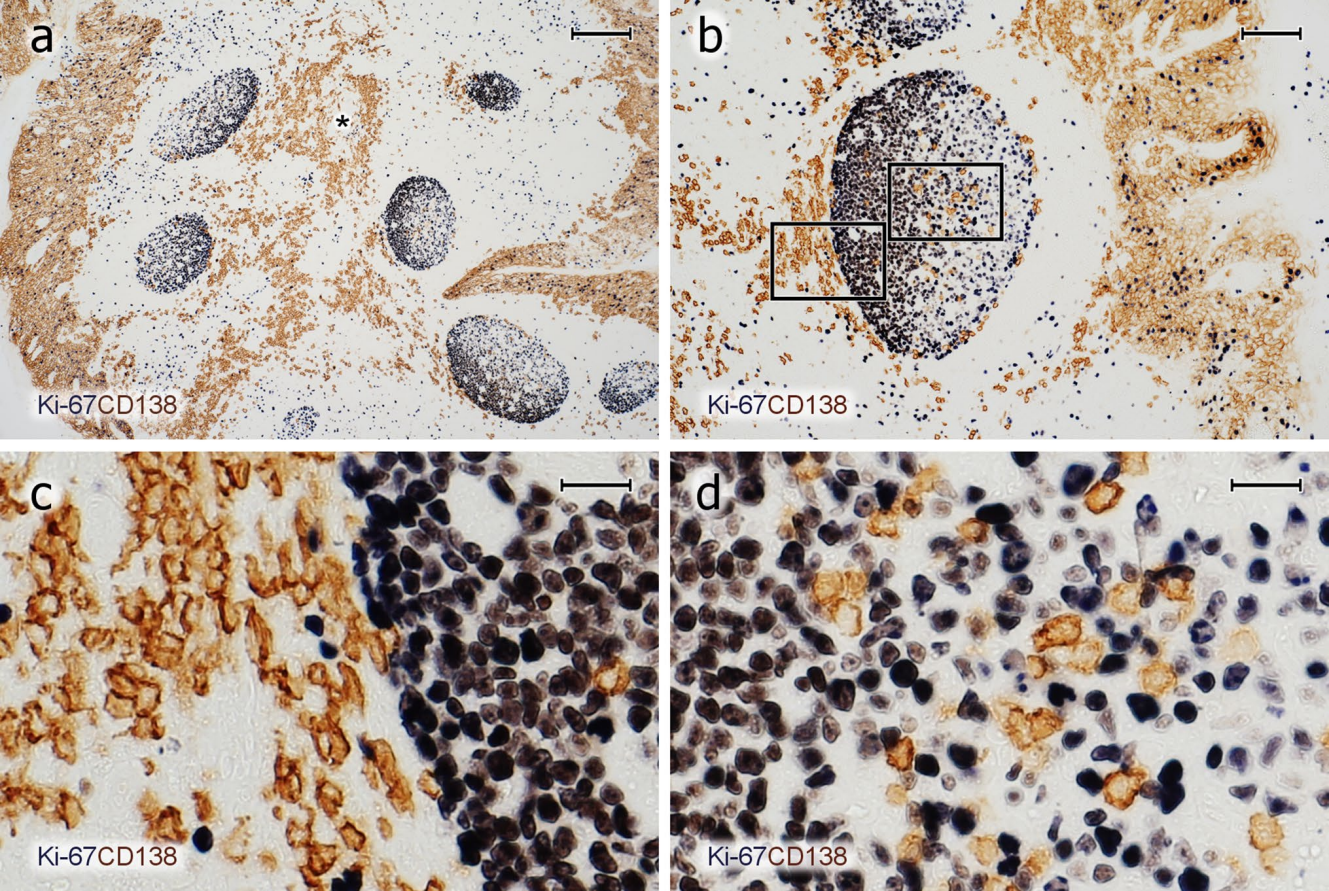
PCs inside and outside GCs do not proliferate. **a**–**d** Visualisation of Ki-67 (mAb MIB-5, blue-black) followed by staining for CD138 (mAb BB-4, brown) shows that the vast majority of PCs inside and outside GCs is Ki-67^−^. **c**, **d** Magnifications of the areas indicated in **b**. The entire crypt epithelium is also CD138^+^ (**a**, **b**). It covers both sides of the specimen in **a** and the right side in **b**. The brown cells in the central part of **a** represent PCs accompanying the central connective tissue septum (asterisk) between two crypts. **a**–**d** 21-year-old female. Scale bars 200 µm (**a**), 100 µm (**b**), and 20 µm (**c**, **d**)

**Fig. 4:**
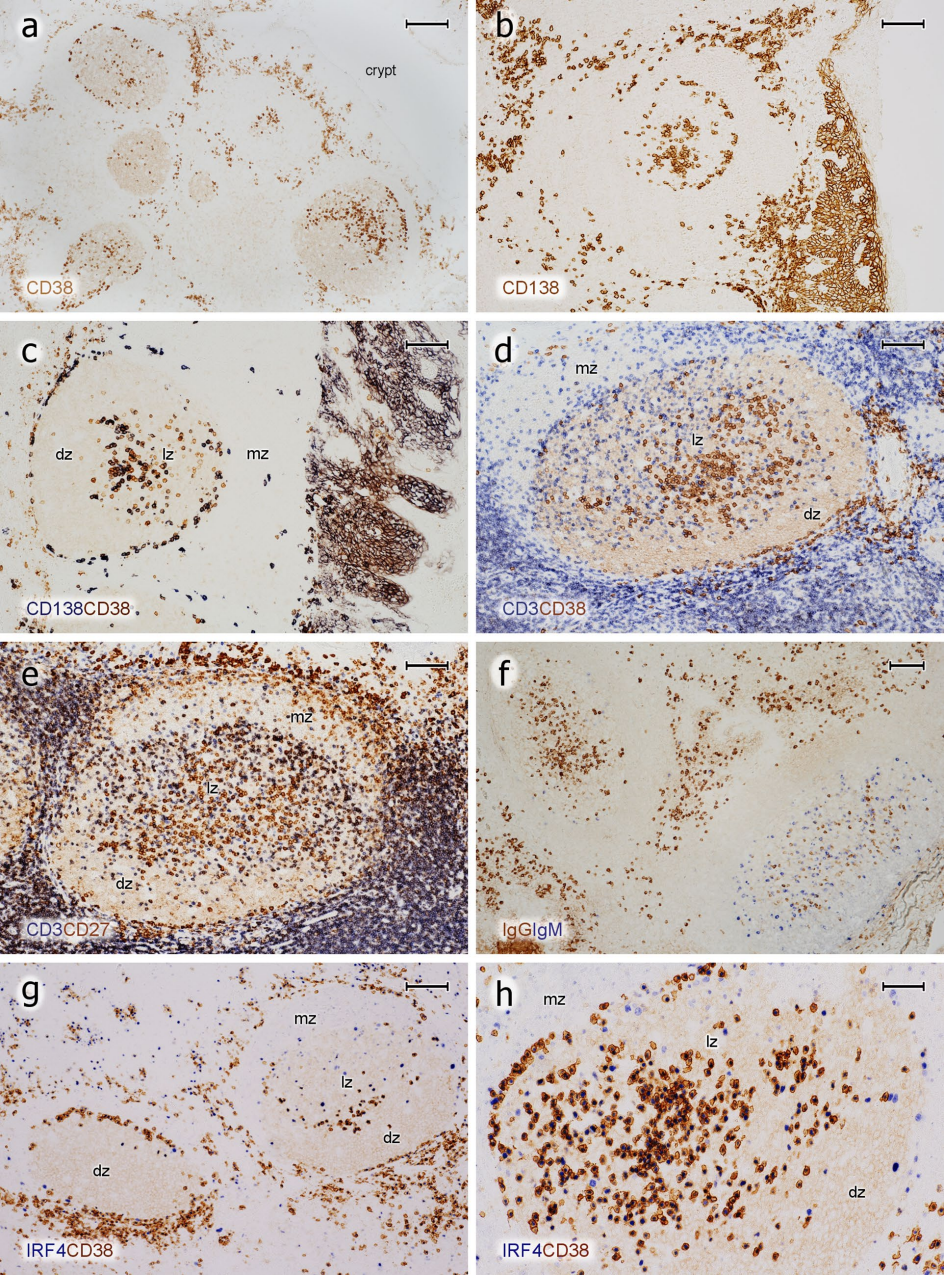
Phenotype of PCs in tonsil GCs. **a** Single staining for CD38 (mAb SPC32) and **b** CD138 (mAb BB-4). The crypt epithelium is located at the left and right margins in **a** and at the right margin in **b**. **c** Staining for CD138 (blue-black) followed by staining for CD38 (brown). **d** Staining for CD3 (polyclonal Abs, blue) followed by visualisation of CD38 (brown). **e** Visualisation of CD3 (dark blue) and subsequently of CD27 (mAb 137B4, brown). CD27^++^ PCs are located beneath the epithelium (upper rim of image) and in the GC. The superficial CD27^+^ cells in the mantle zone adjacent to the subepithelial PCs represent memory B cells. **f** Double staining for IgG (mAb RWP49, brown) and IgM (polyclonal Abs, blue). Two GCs differ in the prevalence of IgG^+^ (left GC) and IgM^+^ (right GC) PCs. The crypt epithelium and subepithelial PCs are hit in the centre and lower left corner of the image, where detritus in a crypt stains dark brown. **g** Overview of two follicles stained for IRF4 (mAb EP190, blue-black) and CD38 (mAb SPC32, brown) (**h**). Additional follicle stained in the same way showing a combination of the PC patterns of the two GCs in **g**. The epithelium is located near the upper or upper left parts of the image in **d**, **e**, **g**, **h**. *mz* mantle zone, *lz* light zone of GC, *dz* dark zone of GC. **a**–**h** 21-year-old female. Scale bars = 200 µm (**a**), 100 µm (**b**–**g**), 50 µm (**h**)

**Fig. 5:**
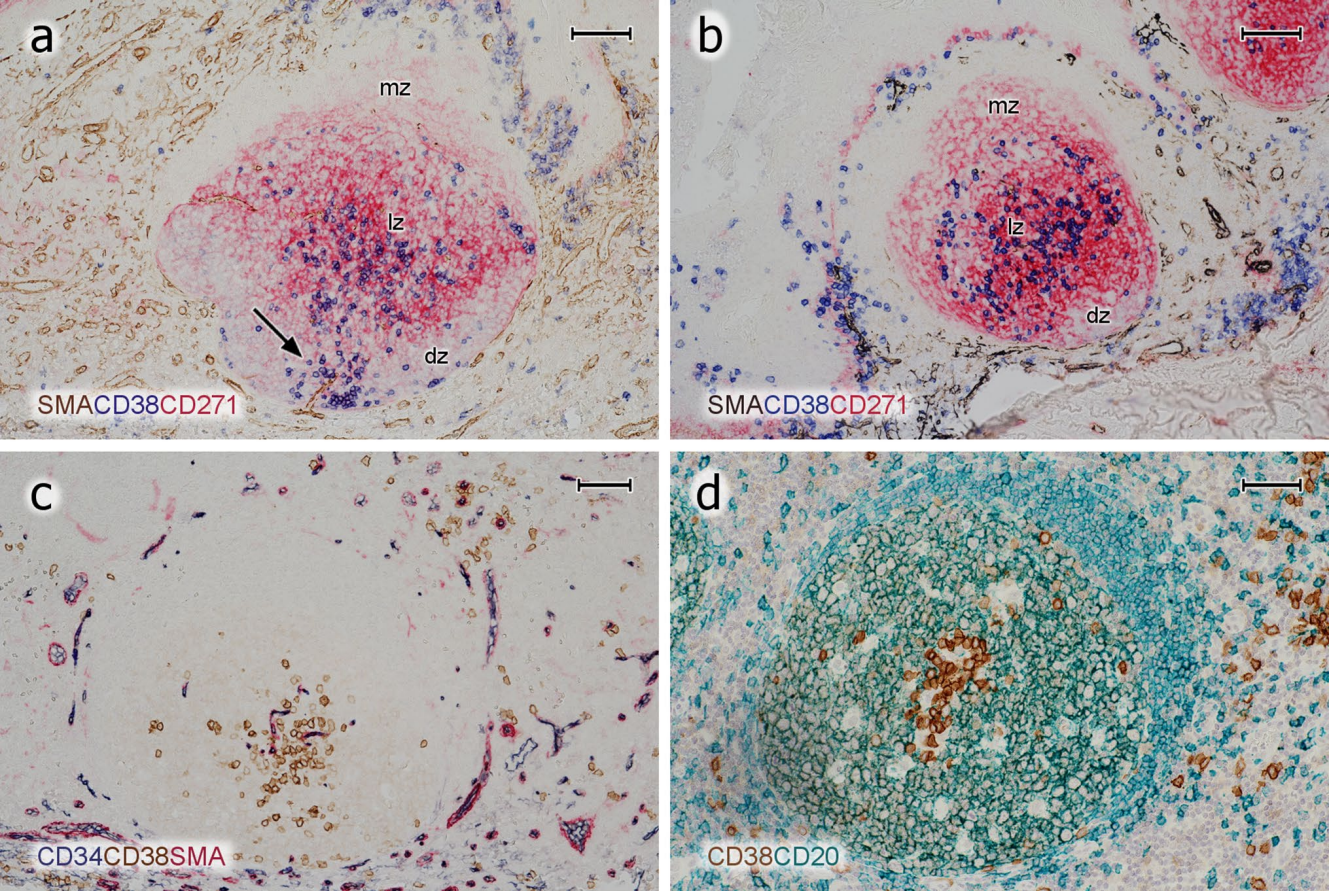
Localisation of PCs, FDCs, and capillaries in GCs. **a** Follicle stained for SMA (polyclonal Abs, brown), CD38 (blue), and CD271 (red). In this exceptional GC, CD38^++^ PCs also appear in the dark zone and accompany a microvessel (arrow). **b** Follicle stained for SMA (brown-black), CD38 (blue), and CD271 (red). CD38^++^ PCs are preferentially located in the basal light zone, where the density of red CD271^+^ FDC processes is highest. **c** Staining for CD34 (mAb QBend-10, blue), CD38 (mAb SPC32, brown), and SMA (red). CD38^++^ PCs often occur loosely distributed in the vicinity of CD34^+^ GC capillary endothelia in the light zone. **d** They may, however, also be directly adjacent to capillaries or perivascular FDCs (CD38 first stained in brown and, subsequently, CD20 in green). *mz* mantle zone, *lz* light zone of GC, *dz* dark zone of GC. **a**, **d** 57-year-old male. **b**, **c** 38-year-old female. Scale bars 70 µm (**a**–**c**), 100 µm (**d**)

Single staining for CD38, CD138, and IRF4 as well as for icIgG or icIgM revealed a conspicuous and relatively numerous strongly positive cell type in most GCs of all individuals tested (Figs. 2a–c, 3a–d, 4a–h). Double staining for CD3 and CD38 or CD138 together with double staining for CD3 and CD27 showed that the strongly staining cells were CD3^−^. They accumulated in the basal light zone (Figs. 2b, 3b, 4a–e) often forming dense clusters (Figs. 2a, 3b, 5c, d), but also occurred scattered in the entire light zone (Figs. 2c, 4h). In addition, the cells often formed linear aggregates at the GC surface towards the mantle zone (Figs. 3b, 4b, c, g, h). We concluded that we were confronted with intra-GC PCs and that CD38, CD138, CD27, and IRF4 were strongly expressed by predominantly—but not totally—overlapping cell populations. In several individuals and in a larger number of GCs, the presumed plasma cells were also located at the outer circumference of the dark zone towards the perifollicular space. They appeared to crawl along well-visible elongated FDC processes and/or connective tissue fibres delimiting the dark zone from the surroundings (Figs. 2a–c, 3b, c, 4c–e, g). In some specimens, these PCs continued from the peripheral dark zone of the GC into the perifollicular connective tissue, thus forming a bridge of cells finally contacting accumulations of PCs around the deep collagenous tissue septum between the crypts (Fig. 3a, b, 4g). After assessing the phenotype of intra-GC PCs, strong expression of CD38 was chosen for detecting these cells. It was, however, clear that a small population of CD38^− (or ±)^icIg^+^ PCs was missed by this strategy (see below). Interestingly, a large number of the plasma cells detected could be identified in hemalum-eosin stained sections because of their strong cytoplasmic staining.

The number and predominant location of intra-GC PCs was somewhat variable among different GCs in the same section. Slight inter-individual variations were also evident. Both phenomena appeared to be independent of differences in sectioning levels with respect to single GCs. In most follicles, PCs were almost absent from the interior of the dark zone and from the mantle zone (Fig. 2a, b, 4a–e, g). However, exceptions from this rule were noted when 300 serial sections of a specimen were inspected. In this specimen, focal linear accumulations of CD38^++^ PCs occasionally occurred in the dark zone and in the mantle zone for about 10–20 serial sections (Fig. 5a). These PCs appeared to be located in the vicinity of blood vessels crossing the dark zone or the mantle zone.

Double staining for CD271 and CD38 or triple staining for SMA, CD38, and CD271 showed that the highest density of FDC processes occurred in the basal light zone, where most PCs were found (Fig. 5a, b). In addition, this area also had the highest density of intra-GC capillaries visualised by SMA^+^ pericytes or by staining for CD34 (Fig. 5c). If PCs formed clusters (Fig. 5c, d), these appeared to be loosely associated with intra-GC capillaries. The PCs did, however, not always directly approach the capillary endothelia, but were found at some distance from the microvessels. This phenomenon appeared to be caused by FDCs enveloping GC microvessels (Fig. 2d–f).

Outside GCs, major numbers of PCs were predominantly found in two locations. First, they occurred within and below the crypt epithelium and, second, PCs were located in and near a deep coarse connective tissue septum between the crypts (Figs. 1b, 3a). This septum appeared to exist of thick parallel collagenous fibres arising from the tonsil capsule and supporting larger vessels and numerous lymphatics. The numbers of PCs in both regions and their predominant icIg isotype were variable among the individuals (see below).

### Detailed phenotype of PCs in GCs

Immunohistology as performed in the present study is not appropriate to reveal details of membrane antigen co-expression, because it is designed as a subtractive staining technique. Only if the location of two antigens is spatially separate, for example, in the nucleus or the membrane, may co-expression be visualised. However, by reversing the sequence of primary antibodies used for double-staining, cells expressing only one of two membrane antigens can be demonstrated. This permits to derive coarse expression patterns of two membrane antigens by comparison to single-stained controls in the same test.

**Fig. 6:**
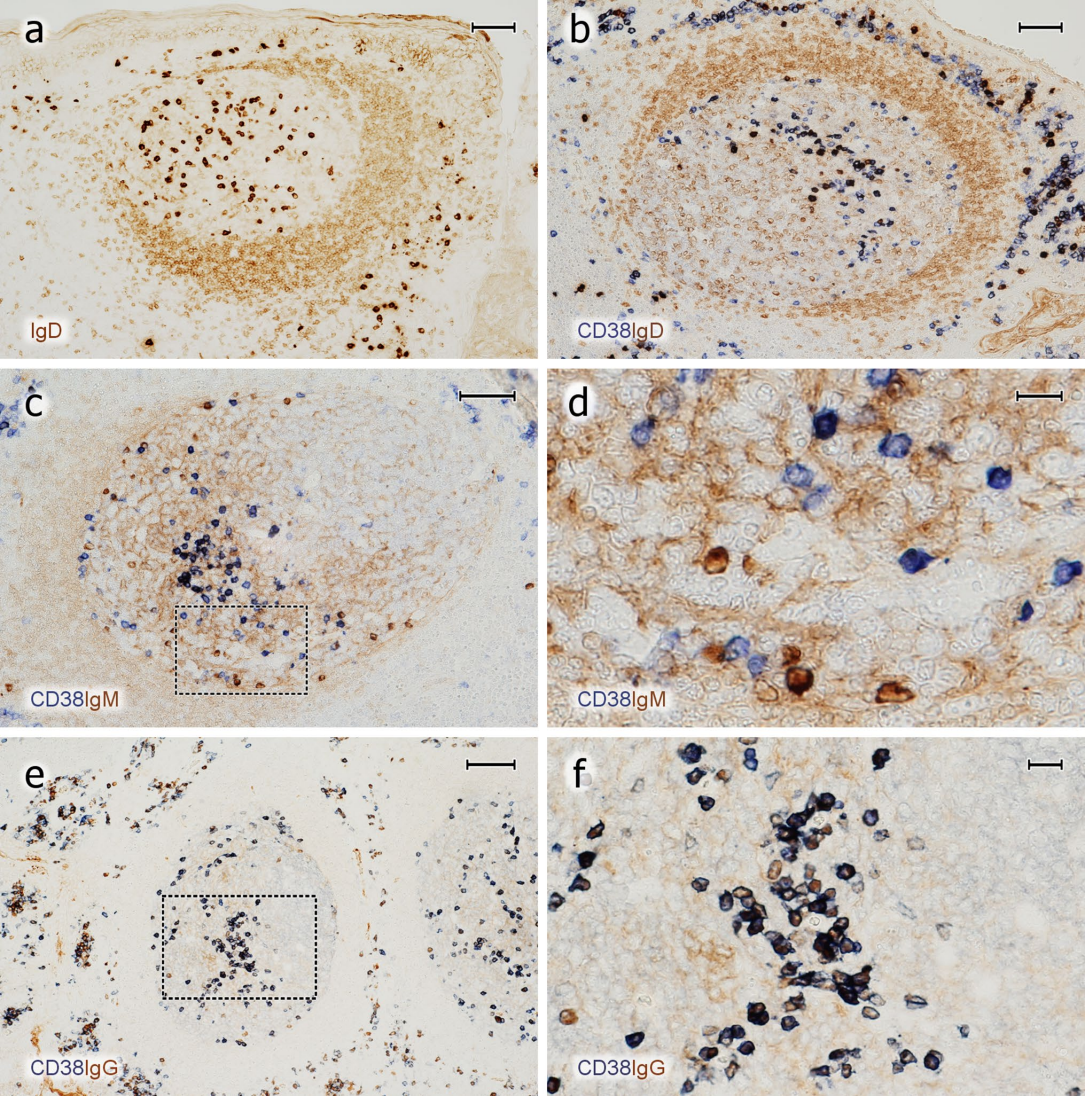
Expression of CD38 by icIg^+^ PCs in GCs. **a** Distribution of surface and icIgD in an icIgD^+^ GC. A minority of GCs contain a large number of IgD^+ +^ PCs and also weakly IgD ^+ ^ cells (cell surface or intracellular staining). The mantle zone (brown, right) is formed by small recirculating surface IgD^+ ^ B-lymphocytes. **b** GC stained first for CD38 (mAb SPC32, blue) and then for IgD (polyclonal Abs, brown). The dark blue PCs may be single or double positive. The entire GC is occupied by weakly IgD^+^ cells. These cells are less intensely stained than the surface IgD^+^ cells in the mantle zone (right). **c** Visualisation of CD38 in blue and icIgM in brown. The mantle zone is directed towards the left part of the image. **d** Higher magnification of area marked in **c**. There are several icIgM^+^ cells with low or absent staining for CD38. Weak surface staining of IgM^+^ mantle zone cells and staining of IgM^+^ immune complexes on FDCs is accepted in **c** and **d** to increase the sensitivity of the method. **e** In contrast to icIgM, staining for CD38 (mAb SPC32, blue-black) and icIgG (mAb RWP49, brown) reveals only very few icIgG^+^CD38^±^ cells in this specimen. The right part of the GC contains the dark zone. **f** Higher magnification of area indicated in **e**. The crypt epithelium is located near the upper margin or upper right margin of the images in **a**–**c** and near the left margin in **e**. **a**–**f** 38-year-old female. Scale bars 70 µm (**a**–**c**), 14 µm (**d**), 100 µm (**e**), and 20 µm (**f**)

The intensity of CD38 expression in intra-GC plasma cells was rather high and most of them were clearly different from centroblasts and centrocytes, which only exhibited very weak CD38 expression. However, the staining intensity of CD38 in plasma cells was not uniform and a minority of the cells were hardly distinguishable from GC B cells. When CD138 and CD38 were stained successively, both populations did not totally overlap. Often, more CD38^++^ PCs were found than CD138^+^ PCs, irrespective of their location in the GC (Fig. 4c). The intensity of staining for CD138 in GCs and in subepithelial plasma cells was rather variable among different individuals. CD38 showed a more consistent expression in different specimens and it was thus chosen as the preferential plasma cell reagent. When icIgs were stained after CD38, it became clear that most CD38^++^ cells in GCs carried icIgG. The next frequent Ig was IgM followed by IgD (Fig. 6a) and IgA. However, in one of the six individuals, the numbers of icIgG^+^ and icIgM^+^ PCs seemed to be almost equal in GCs. In this individual, single GCs appeared to differ in the relation of icIgG to icIgM PCs (Fig. 4f). In general, IcIgD^+^ PCs were even more variable in different GCs (see below). Small numbers of icIgM^+^, icIgD^+^, or icIgA^+^ PCs in GCs (and in the intra/subepithelial region) revealed only faint or absent expression of CD38 (Fig. 6b–d). Inside and outside GCs, strong expression of CD38 was more highly correlated to intracellular staining for IgG than to any other Ig (Fig. 6e, f). Thus, strong expression of CD38 may be a relatively late event in PC development.

In summary, it was deduced that the majority of intra-GC PCs expressed CD38, CD27, and CD138, although PCs differed in the percentage of cells positive for each antigen (not shown) both among GCs and among individuals.

### PCs and Ki-67 in GCs

The most surprising finding was that Ki-67 was undetectable in the majority of cells strongly positive for CD38 and/or CD138 or for icIgs. This was true for all six individuals investigated. Thus, we classified these cells as plasma cells and not as plasmablasts. This result did not seem to be due to technical problems of double-staining, because it was true both for cell membrane and for intracellular antigens. In addition, co-expression of Ki-67 was easily visualised in B cells together with CD30 or AID (see below).

**Fig. 7:**
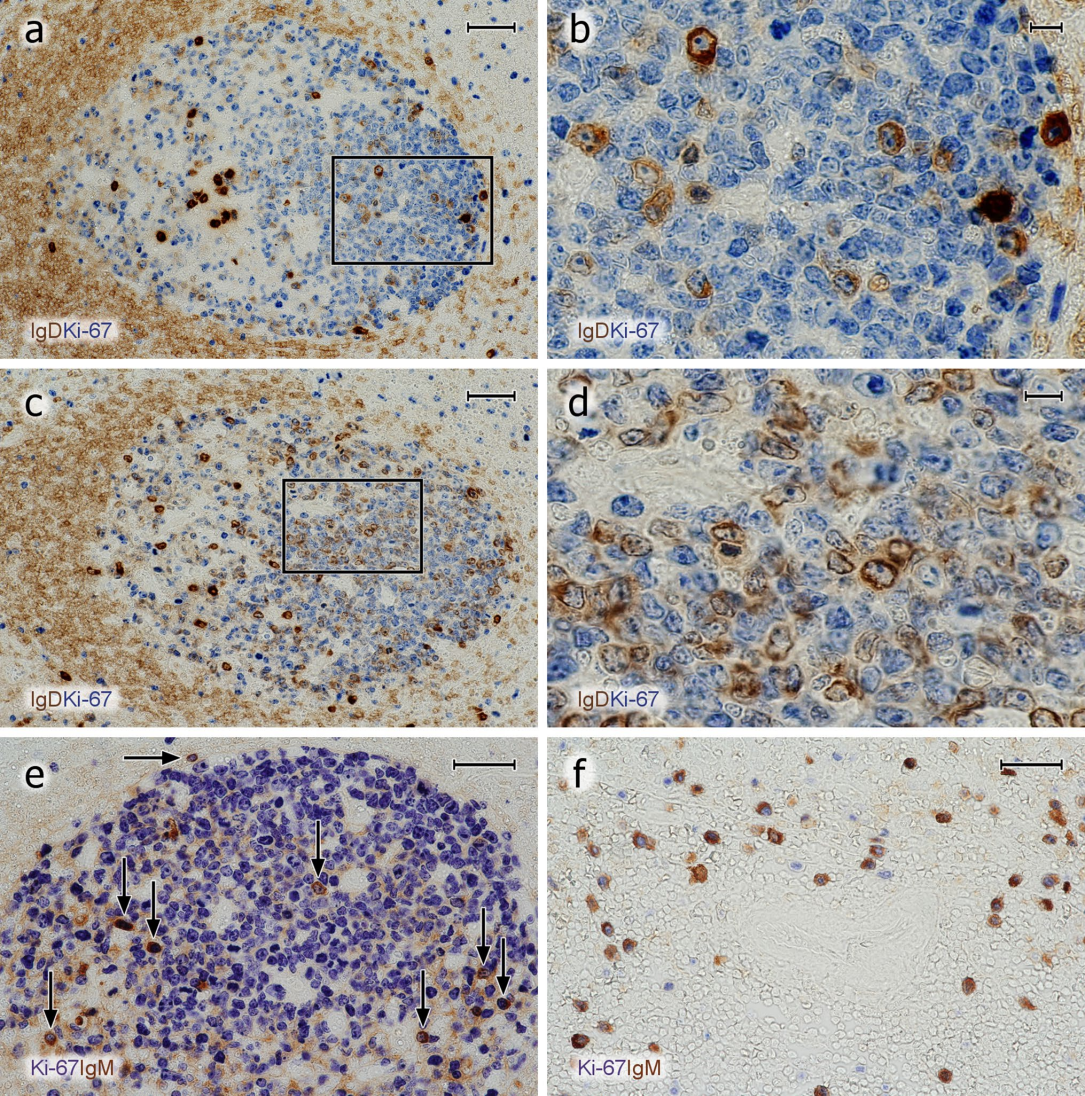
Proliferation of icIg^+^ cells. **a**–**d** Visualisation of IgD^+^ cells (polyclonal Abs, brown) followed by detection of Ki-67 (mAb MIB-5, blue). **a**, **b** There are several Ki-67^+^icIgD^+^ plasmablasts in the dark zone and Ki67^−^icIgD^++^ PCs in the basal light zone. The icIg^+^ cells in the dark zone do not exhibit the typical nuclear morphology of PCs. **c**, **d** In another follicle of the same individual, even more weakly IgD^+^ cells with Ki-67 expression are seen in the dark zone. It is impossible to distinguish intracellular and surface staining. Such GCs are infrequent. **e** Several dark zone Ki-67^+^ cells stained blue in the first step show icIgM in the second step (brown) of staining (vertical arrows). Single double-positive cells always occur at the surface of the dark zone (horizontal arrow). **f** In the outer T-cell region of a human spleen Ki-67 (blue) is demonstrated in about 50% of icIgM^+^ migratory plasmablasts (brown). The unstained structure in the centre of the image is a bending central artery. Same method as in **e**. The crypt epithelium is located beyond the left margin in **a**, **c** and below the bottom of the image in **e**. **a**–**e** 38-year-old female. **f** 17-year-old male. Scale bars 50 µm (**a**, **c**), 10 µm (**b**, **d**), and 40 µm (**e**, **f**)

A few Ki-67^+^ GC cells with icIg, i.e., plasmablasts, were detected after double-staining for Ki-67 and IgM, IgD, IgG or IgA (Fig. 7a–e). IgE-positive B lymphocytes or PCs were not demonstrable by the reagent applied. The icIg^+^Ki-67^+^cells were scattered in the entire GC with some preference for the basal light zone. When IgD was excluded, the largest number of Ki-67^+^ plasmablasts was found with anti-icIgM. Anti-icIgG and anti-icIgA only revealed very few plasmablasts; in most cases, 1–4 Ki-67^+^ cells per GC section. Single icIgM^+^Ki67^+^ plasmablasts were also found at the surface of the dark zone (Fig. 7e). The frequency of icIgM^+^Ki-67^+^ cells appeared to be higher than that of CD38^++^Ki-67^+^ cells, which may indicate that CD38 is associated with more mature PCs and may not detect all icIgM^+^ plasmablasts. IgD was special, because, as published previously (Arpin et al. 1998), the number of intra-GC icIgD^+^ plasma cells was extremely variable among different GCs in the same individual and also among different individuals. In some individuals, icIgD was almost absent from all GCs, while other individuals showed several GCs with large numbers of icIgD^+^ PCs. IgD cell surface staining is well detectable by immunohistology, because this Ig is of rather low abundance in the interstitial space. This fact permitted observing low quantities of icIgD (and perhaps also cell surface IgD) in many centroblasts in a minority of specimens and GCs. Sometimes, even centrocytes were found to express minor amounts of cell surface or icIgD (Fig. 7a–d). In contrast to other Igs, major numbers of Ki-67^+^ cells staining more or less strongly for icIgD were observed in the dark zone of GCs harbouring icIgD^+^ PCs. However, in weakly positive cells, it was impossible to diagnose whether IgD was intracellular or on the cell surface (Fig. 7b, d). Thus, there appeared to be a continuous transition from centroblasts with membrane or icIgD to plasmablasts.

In summary, with the exception of IgD in some GCs, the number of intra-GC plasma cells vastly exceeded that of intra-GC Ki-67^+^ plasmablasts detectable by staining for icIg in all tonsils. In GCs, IgM was the predominant icIg associated with Ki-67. In contrast to PCs, the few plasmablasts positive for icIgM, icIgG, or icIgA did not occur in special areas of the GC (Fig. 7e). It is unlikely that the apparent lack of GC plasmablasts was caused by technical problems, because the same staining protocol detected large numbers of Ki-67^+^icIgM^+^ plasmablasts in the recirculation compartments of the white pulp, i.e., in the outer T-cell regions and at follicular surfaces (Fig. 7f), in some—but not all—adult spleens.

### PCs and plasmablasts in the intra-/subepithelial regions and near the deep connective tissue septa

IcIgA and icIgG tended to represent the predominant isotypes in intra- and subepithelial PCs. However, there were two individuals in whom intra-/subepithelial icIgD PCs were more frequent than icIgA and icIgG PCs. Subepithelial PCs tended to be larger than the plasma cells found in GCs and exhibited more regular round nuclei. In all individuals, the majority of the intra-/subepithelial icIg^+^ cells were large oval Ki-67^−^ PCs. Only cells with less icIg and a relatively round shape occasionally exhibited Ki-67^+^ nuclei. However, one individual exhibited a higher number of large icIgD^+^Ki-67^+^ cells of typical PC morphology. Near the deep connective tissue septa between crypts, most PCs contained icIgG in all individuals. The second-most frequent isotype in this location was IgA.

In contrast, only very few icIgM^+^ PCs were visible near epithelia or near the connective tissue septa. Cells exhibiting this isotype were primarily found in GCs. They also occurred in the perifollicular and parafollicular regions in very low numbers. Among icIgM^+^ cells in this location, high numbers of small icIgM^+^Ki-67^+^ plasmablasts were present.

Thus, PCs of each Ig isotype had a characteristic distribution pattern. IcIgM^+^ PCs were rare outside GCs, while icIgA^+^ and icIgG^+^ PCs were most often found in intra- and subepithelial regions. However, in two individuals, IcIgD^+^ PCs represented the most frequent cell type in these regions. IcIgG^+^ PCs also prevailed near the deep connective tissue septa (Fig. 1b).

### Plasma cells in GCs of other lymphatic organs

**Fig. 8:**
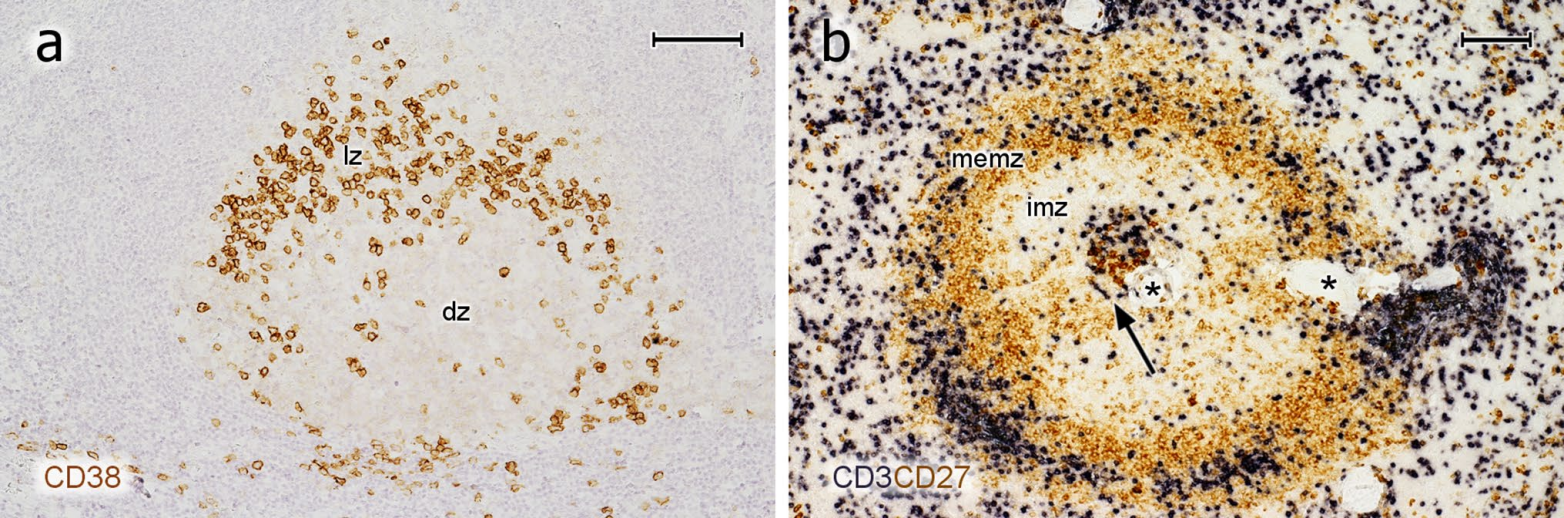
PCs in lymph node and spleen GCs. **a** Single staining for CD38 in a follicle of a cervical lymph node. The distribution of CD38^++^ PCs is similar to that in tonsils (compare Fig. 4a). *lz* light zone of GC, *dz* dark zone of GC. The mantle zone cannot be delineated. **b** Double staining for CD3 (dark blue) and CD27 (brown) in an adult human spleen. This method is optimal to visualise all regions of typical degenerated secondary follicles in normal human spleens (Steiniger et al. 2005, 2006). The follicle consists of a very small GC populated by CD27^++^ PCs and T cells (arrow), a broad inner mantle zone (imz, unstained), and a CD27^+^ memory B-cell zone (memz), which consists of the outer mantle zone and a superficial zone (not visualised in detail). Major numbers of T cells also occur in the superficial zone. Asterisks indicate a follicular artery. **a** 58-year-old male. **b** 21-year-old female. Scale bars 200 µm (**a**), 100 µm (**b**)

Intra-GC plasma cells were not only found in tonsil GCs, but were also present in lymph nodes and certain spleens. Preliminary investigations showed these cells in a cervical (Fig. 8a) and a mesenteric lymph node and in several "normal" human spleens removed because of blunt abdominal trauma (Fig. 8b). The GCs in these spleens were rather small and non-polarised (Steiniger et al. 2011). In spite of this, the phenotype of PCs in splenic GCs was roughly similar to that found in tonsils. Thus, intra-GC PCs occur in different human lymphatic organs irrespective of inflammation.

### AID inside and outside GCs

To further differentiate B lymphocytes in GCs, we visualised AID and CD30. These antigens were chosen, because we wanted to obtain an overview, which types of B lymphocytes existed in the outer zone at the surface of GCs in addition to the PCs described. AID and CD30 had been reported by others (Cattorettti et al. 2006, Fallini et al. 1995, Greiner et al. 2005) to be predominantly, but not exclusively, expressed by B lymphocytes in this area. Both antigens were additionally found in B lymphocytes outside tonsil GCs (Cattoretti et al. 2006; Marafioti et al. 2003; Weniger et al. 2018), and thus, they need to be described in both locations.

With respect to AID, we used two different mAbs (Table 1) and different detection systems such as an alkaline phosphatase-conjugated polymer system, a two-step indirect peroxidase method, the biotin-based ABC system, and a highly amplifying ABC/tyramide detection. We did not try to specifically localise AID in cell nuclei, which may only be possible with less amplification (Cattoretti et al. 2006), but we wanted to detect the overall distribution of AID in different cell types.

**Fig. 9:**
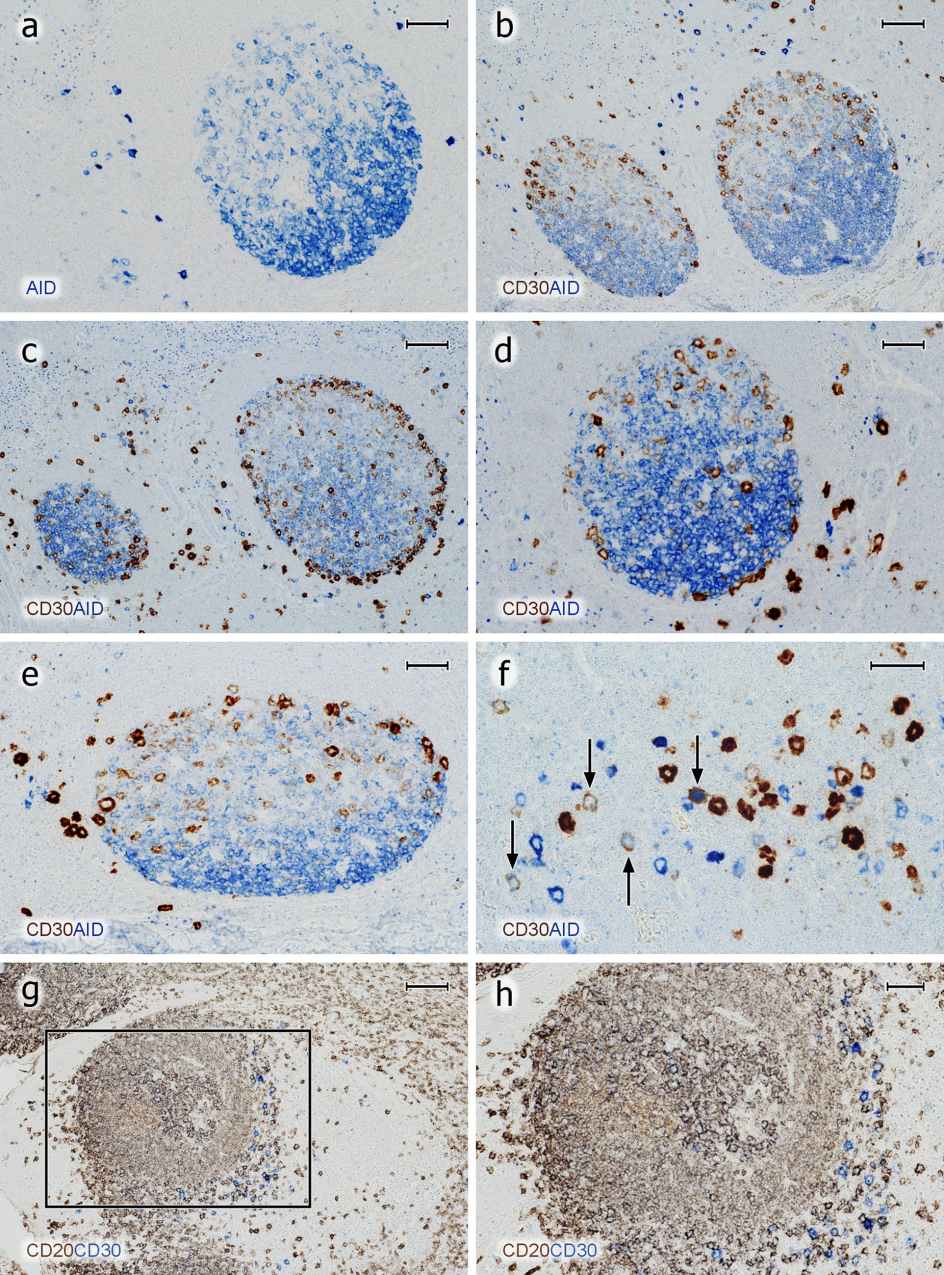
AID^+^ and CD30^+^ B cells inside and outside GCs. **a** Distribution of diffuse intracellular staining for AID (mAb ZA001, blue) inside a GC and strong reactivity in single extrafollicular B cells. The granular staining observed in the upper left and right corner is located in plasma cells and may represent inactive AID in the cytoplasm. **b** Demonstration of CD30 (mAb Ber-H2, ABC-tyramide method, brown) and AID (mAb ZA001, BrightVision AP polymer with Enzo HighDef blue for AP, blue). CD30^+^ GC cells exhibit increasing staining intensity towards the surface of the light zone. In addition, CD30^++^ and AID^++^ cells occur in extrafollicular areas. **c** In a few GCs, but not in all, CD30^+^ cells (brown) may also occur at the surface of the dark zone. **d** CD30^+^ cells (brown) appear to crawl along the surface of the dark zone. **e** The CD30^+^ cells (brown) at the GC surface appear to continue towards the perifollicular CD30^++^ cells (dark brown) in the T/B area. **f** The majority of the CD30^++^ and the AID^++^ extrafollicular cells are single-positive, but double-positive cells also occur (arrows). Most of these cells are weakly stained for both antigens. **g** Follicle stained for CD20 (mAb L26, brown) and subsequently for CD30 (mAb Ber-H2, blue). The CD30^++^ cells in the perifollicular area are CD20^±^ or CD20^−^. The unstained areas represent the crypt epithelium (left) and a T-cell region (right). **h** Higher magnification of area indicated in **g**. The method mentioned in **b** was also used in **c** to **d**. In **a** to **e,** the crypt epithelium (not depicted) is located above the GC. **a**–**h** 38-year-old female. Scale bars 50 µm (**a**, **d**, **e**), 100 µm (**b**, **c**, **g**), 40 µm (**f**), and 50 µm (**h**)

MAb ZA001 produced a somewhat stronger staining than mAb EK2 5G9 with the systems used. Using mAb ZA001, granular intracellular staining for AID was detected in multiple cell types in all organs investigated including hepatocytes and other epithelial cells. Dot-like intracellular staining was also observed in the majority of the intra- and subepithelial plasma cells (Fig. 9a–d) and also in CD38^++^ plasma cells in GCs. These dots were vastly enlarged after tyramide amplification. Small intracellular dots also occurred in lymphocytes in T-cell regions and in mantle zones. We thus concluded that dot-like staining did not correlate with active AID, but might represent complexes of sequestered inactive enzyme in the cytoplasm. The only cells which exhibited diffuse intracelluar AID staining were centroblasts and large single cells in the perifollicular T/B area (Fig. 9a–f). On more detailed inspection, centroblasts did not only stain diffusely, but also exhibited dot-like AID staining in their cytoplasm. AID^+^ cells filled the dark zone and tended to superficially extend around the light zone. This arrangement often provoked a dish-like shape of the dark zone, because the diffusely AID-positive cells did not surround the entire circumference of the light zone, leaving out its apical pole (Fig. 9a). Sometimes, a central elevation appeared to be present in the dark zone area of the dish. Outside follicles, isolated AID^+^ cells were the most strongly stained cells present in tonsils (see below). The vast majority of these cells were also positive for CD20 and nearly all of them exhibited Ki-67^+^ nuclei. Very few AID^+⁠+^Ki67^−^ cells were, however, also observed in the T/B area. Thus, most of the extrafollicular AID^++^ cells obviously represented B-cell blasts.

### CD30 inside and outside GCs

Using mAb Ber-H2, we found that CD30^+^ B cells in GCs were most easily detected in cryosections. In paraffin sections, tyramide amplification had to be applied to optimally visualise these cells, which may indicate that the epitope detected is sensitive to formalin fixation. There were extreme individual differences in the number and staining intensity of CD30^+^ cells in tonsils. In GCs, CD30^+/++^ large cells were found in a superficial position primarily in the light zone (Fig. 9b), but in a number of GCs, these cells additionally formed a chain of cells also at the surface of the dark zone (Fig. 9c, d). The vast majority of these CD30^+/++^ cells were Ki-67^+^ and CD20^+^. In addition, weakly CD30^+^ cells also occurred deep inside the light zone. In fact, increasing CD30 expression was observed from the interior to the surface of the light zone. When using cryosections, some cells weakly expressing CD30 were additionally present inside the dark zone of GCs in some, but not all, individuals. Double-staining for AID and CD30 at the surface of GCs was inconclusive, because AID gave only relatively weak intracellular staining. About half of the CD30^+/++^ cells at the surface of the light zone showed intermediate or strong nuclear reactivity for IRF4. The fraction of double-positive cells was individually variable. When AID and CD30 were compared in paraffin sections, the overall distribution of both antigens in GCs appeared complementary. CD30 prevailed at the surface of the light zone, while AID was primarily found in the dark zone, although several AID^+^ cells with diffuse intracellular staining were also distributed in the light zone (Fig. 9a–e).

The CD30^++^ cells at the surface of the light zone seemed to be larger than the AID^+^ B cells in the dark zone and at the GC surface approaching the light zone. However, membrane staining always causes cells to appear larger, and thus, the phenomenon may be a general feature of membrane versus intracellular reactivity.

Outside GCs, a population of large CD30^++^ cells was found in the perifollicular mixed T/B areas and in the T-cell zones (Fig. 9c–h). These cells stained more strongly for CD30 than the superficial cells of the GC light zone. In several GCs, CD30^+/++^ cells appeared to detach from the GC surface at the dark zone (Fig. 9c, d) or at the surface of the border between light zone and dark zone (Fig. 9e) to approach the extrafollicular CD30^++^ cells. However, in one individual, the entire area outside the GC, including the mantle zone, was filled with CD30^++^ cells.

The number of extrafollicular CD30^++^ cells tended to be low and differed among individual specimens. In some individuals, all of these cells were IRF4^+^. In general, the expression intensity of CD30 and nuclear IRF4 appeared to be correlated (not shown). Our technique did not permit a detailed investigation of CD30 and CD20 in extrafollicular cells, but it was apparent that a part of the CD30^++^ cells was CD20^±^ or even CD20^−^ (Fig. 9g, h). CD30^++^ cells were CD3^−^ and thus did not represent T cells. Double staining for AID and CD30 showed that some individuals had a large number of extrafollicular AID^++^CD30^−^ cells and a minor population of double-positive cells (Fig. 9f). AID^−^CD30^++^ cells were not assessed in detail, but the number of CD30^++^ cells appeared to exceed that of AID^++^ cells (not shown). The vast majority, but not all, of the extrafollicular CD30^++^ cells were Ki-67^+^.

## Discussion

The results reported in this study show that large numbers of PCs are present in special areas of human tonsil GCs. This information permits a new hypothesis on the origin of at least a part of the local PCs in tonsils. In addition, it indicates the necessity for precise spatial analysis at the mesoscopic scale. This may be done by reconstructing entire follicles and their PCs in three dimensions using immunostained serial sections.

PCs are predominantly located in the border area of the GC light zone close to the dark zone, i.e., in the basal light zone. In GCs, most PCs contain icIgG, but individually variable numbers of PCs expressing icIgM, icIgD, or icIgA are also present. IcIgD^+^ PCs are only found in a minority of GCs, the frequency of which is individually variable. The overwhelming majority of the Ig-containing cells do not proliferate and, thus, do not represent plasmablasts, but correspond to more or less mature plasma cells. Most of the PCs express typical PC cell membrane antigens such as CD38, CD27, and CD138, but their individual phenotype is somewhat variable. They all stain for nuclear IRF4. Only very minor numbers of Ki-67^+^ plasmablasts with icIg are found in tonsil GCs. The only exception are IgD^+^ cells in the few GCs containing major numbers of icIgD^+^ PCs.

The basal light zone of GCs has the highest density of capillaries, FDC processes and—in consequence—of deposited antigens. It cannot be excluded that the final maturation of PCs is initiated by these special surroundings (Ise et al. 2018; Kräutler et al. 2017). Pericapillary FDCs might be attractive for PCs and may play a role in this process. In a more general context, FDCs in the basal light zone could not only influence PC differentiation, but could also contribute to GC polarisation by establishing gradients of antigen collected from two different directions, namely, first, from the crypt epithelium via the adjacent lamina propria and, second, from intra-GC capillaries via endothelial transcytosis.

The phenotype of the GC PCs we describe corresponds to that known for human PCs (Khodadadi et al. 2019; Mei et al. 2007; Klein et al. 2006). Indications that IRF4^+^ PCs occur in human tonsil GCs have previously been published by Fallini et al. (2000). A detailed analysis of these cells, their distribution, and their proliferative behaviour has, however, been lacking up to now. The unexpected finding in our investigation is the low number of proliferating plasmablasts in tonsil GCs. There were only few cells with icIgM, icIgG, or icIgA, and expression of Ki-67 in GCs or in other locations, such as the crypt epithelium, the lamina propria below the epithelium, or the deep connective tissue septa between the crypts. This was also true for Ki-67 and cells with high expression of CD38, CD138, and CD27. In the locations mentioned, the highest number of icIg^+^Ki-67^+^ cells was found for IgM and the lowest for IgG. IgD formed an exception, because surface and/or intracellular IgD seemed to be visualised in centroblasts and centrocytes of some GCs. A relatively large number of these cells was Ki-67^+^. Thus, visualisation of IgD may yield the most relevant information on the relation of plasma cells and plasmablasts in GCs. It is very likely that the IgD^+^ cells detected have switched to IgD. Tonsillar IgD-only centroblasts, B cells, and PCs have been described and analysed in some detail using cell suspensions (Liu et al. 1996; Seifert et al. 2009; Arpin et al. 1998). These cells have deleted the Cµ gene, exhibit an extensive number of somatic mutations in their IgH and Igλ genes for IgD, form clones of large size, and exhibit a biased V_H_ gene usage (Liu et al. 1996; Seifert et al. 2009). It has been speculated that this cell type may be induced by superantigens from microorganisms in the oral and nasopharyngeal cavities during extended GC reactions (Seifert et al. 2009). Thus, subepithelial icIgD^+^ PCs may form a decisive part of mucosal protection (Gutzeit et al. 2018). It cannot be excluded that the dynamics of GCs with a high amount of IgD^+^ cells differs from other GCs.

Some general technical aspects have to be considered with respect to visualisation of icIg in plasmablasts. IgD is detected with high sensitivity in paraffin sections, because this isotype has a very low interstitial concentration. Thus, co-staining of ubiquitous interstitial Ig or of immune complexes on FDCs does not pose a problem. This permits detection of a larger number of surface IgD^±^Ki-67^+^ cells in the dark zone of certain GCs together with relatively few icIgD^++^Ki-67^+^ plasmablasts. Combined surface and icIg staining is also possible for IgM. The anti-IgG and anti-IgA reagents used do, however, need relatively high dilutions to avoid co-staining of normal interstitial Ig or immune complexes on FDCs. These dilutions are typically below the detection limit of surface Ig on naive B cells. In this vein, visualising icIgG was only achieved with an especially "ineffective" reagent, mAb RWP49. We suspect that this reagent has a relatively low affinity. Thus, the icIg^±^ population of IgG and IgA plasmablasts may have remained undetected in our investigation. The same phenomenon may also be true for Ki-67 and cells weakly expressing CD38, CD138, and other PC surface antigens. Indeed, cryosections showed slightly more CD38 expression in GCs than paraffin sections. The former type of sections can, however, not be used for visualisation of icIg, because soluble intracellular antigens are often removed during staining of cryosections due to insufficient fixation.

In spite of these caveats, we regard the techniques applied as adequate, because a spleen specimen from an individual with a potential acute B-cell reaction was used as a positive control. In this specimen, large numbers of Ki-⁠67^+^icIgM^+^ plasmablasts were easily detected in the B-cell migration compartments of the white pulp.

We thus assume that the low number of plasmablasts may reflect a crucial phenomenon in chronically persisting GCs, which is due to the presence of PCs and is potentially exaggerated by the technical aspects mentioned. If PCs, indeed, secrete Igs inside GCs, they may be involved in downregulating plasmablast development. Intra-GC PCs might represent a so far unrecognised contribution to Ig affinity maturation. The exchange of pre-existing Ig in immune complexes on FDCs for newly secreted Ig of higher affinity may be a means of promoting antibody affinity maturation in chronic GC reactions. Indeed, feedback mechanisms of systemically injected antibodies in GCs have been demonstrated in mice (Zhang et al. 2013). Up to now, it has, however, never been considered that the exchanging antibodies do not necessarily come from the circulation, but that they may also be locally produced inside GCs and may thus establish an ultra-short feedback loop. Such a mechanism might decisively prevent most newly mutated B cells from surviving up to the plasmablast stage. The selection kinetics for IgD^+^ GC cells may be different and permit more centroblasts to differentiate into plasmablasts.

Whether and how PCs in the interior and at the surface of GCs are related is unknown. We speculate that the PCs at the surface of a GC may either be destined for immediate emigration or may complete several rounds of crawling along the most superficial FDCs. Our findings indicate that PCs finally leave the follicle via the superficial dark zone (see below). We cannot totally exclude that PCs are also able to use other emigration routes, for example in the vicinity of vessels which breach the dark zone or the mantle zone. Such pathways are, however, relatively rare. The final destinies of the PCs generated in chronic tonsil GC reactions may be the subepithelial lamina propria and/or the deep connective tissue septa separating the crypts. This deep connective tissue appears as a functional, but not morphological, equivalent of lymph-node medullary cords. It contains a network of lymphatics which finally drains secreted Ig or emigrating lymphocytes into the circulation.

At least a part of the PCs in GCs may be long-lived, irrespective of whether they are residential or destined to leave the GC. Ki-67 may be preserved for some days after mitosis (Pitcher et al. 2002). Thus, the icIg^+^Ki-67^−^ PCs in GCs are likely to be relatively far away from their last division. The fact that strongly reduced non-polarised GCs in normal adult human spleens often contain several plasma cells (Fig. 8b) is also compatible with PCs staying in GCs for longer times. It is unlikely that these PCs have been produced in splenic GCs at such a stage. However, it cannot be excluded that migratory plasmablasts from other sites are even attracted by highly regressed GCs.

Our results provide the new information that PCs accumulate inside but also at the surface of GC light zones (i.e., in the outer zone) of tonsils, and also of other secondary lymphatic organs. It has been noted previously, that the superficial area differs from other parts of the GC in several aspects. The surface of the light zone is known to be occupied by most of the CD4^+^ T_fh_ cells, but also by CD30^+^ large proliferating B cells. We also confirm that a superficial extension of AID^+^ blasts from the dark zone is present in this location. Furthermore, in some individuals, but not in all, FDCs of the outer zone lack CD23 (Brachtel et al. 1996; Steiniger et al. 2011) and often stain less intensely for the unknown antigen detected by mAb CNA.42 (Steiniger et al. 2011). C-myc has also been reported to be primarily found in the nuclei of cells located in a superficial position in the light zone. A part of these nuclei with intermediate or strong reactivity can be attributed to CD30^+^ B lymphoblasts as described by Cattoretti (2013).

**Fig. 10:**
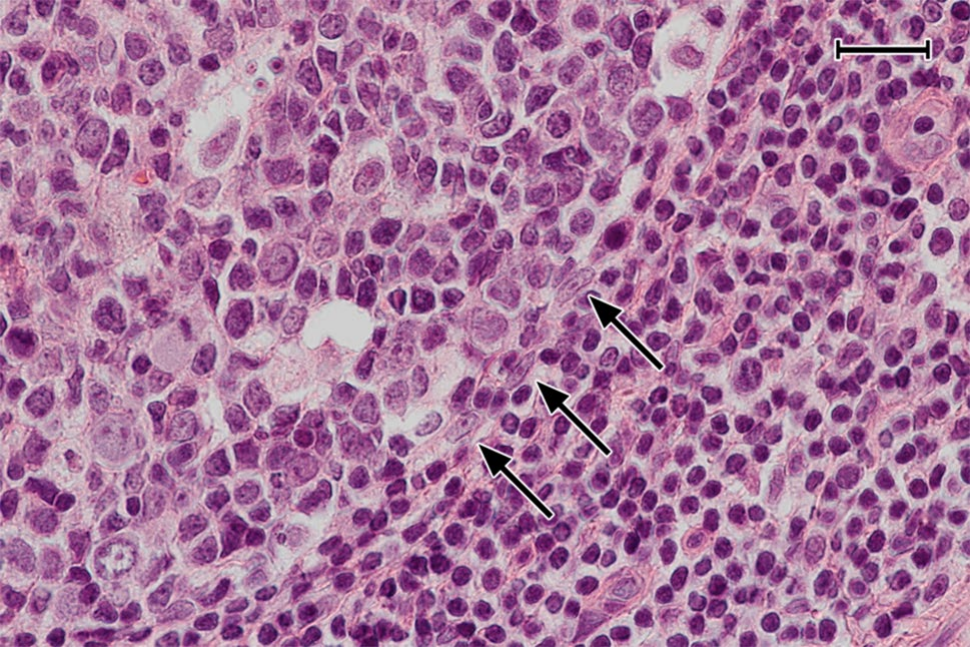
FDC-like cells delimiting the GC towards the mantle zone. Three cells with elongated nuclei and prominent nucleoli (arrows) seem to form a border at the surface of the dark zone of a GC. Routine HE-staining. 38-year-old female. Scale bar 40 µm

In the context of this information, we chose to include CD30 and AID into our investigation to analyse the only B-lymphocyte populations, which have been described to occur in the outer zone of GCs in addition to the PCs found in the present report. The arrangement of the three cell types supports the idea that the outer zone of GCs may be a kind of migration compartment, where gradients of different mediators and/or adhesion molecules guide migratory lymphocytes at an advanced differentiation stage. It cannot be excluded that the most superficial FDCs forming the GC border towards the mantle zone represent a specialised subpopulation of adhesive (or attractive) stromal cells. In hemalum-eosin-stained sections, pale elongated nuclei with a well-visible nucleolus are often observed in this location (Fig. 10).

With respect to AID expression in tonsil GCs and elsewhere, our results correspond to those of others (Cattoretti et al. 2006; Greiner et al. 2005). Utilising highly amplifying immunohistological detection systems, we did, however, find that only diffuse intracellular staining for AID was likely to indicate the presence of an active enzyme. Many cell types (small lymphocytes, centrocytes, plasma cells, hepatocytes, etc.) were found to exhibit one or more AID-positive intracellular granules. We interpret this granular pattern as complexes of AID and RNA or proteins, such as HSP90, which serve to inactivate AID in the cytoplasm (Smith 2017; Methor et al. 2015; Patenaude et al. 2009). Granular complexes were also found in centroblasts together with diffuse staining for AID.

The overall distribution of CD30 in the lymphatic system has been described by others (Cattoretti et al. 2006; Fallini et al. 1995; Schwarting et al. 1989; Marafioti et al. 2003). We speculate that CD30 may be induced by a soluble mediator in GCs, because there is a gradient of CD30 expression in the light zone of the GC with lightly stained cells inside and strongly stained cells at the surface. In rare individuals, weak CD30 expression is also present in certain cells of the dark zone if cryosections are used. AID and CD30 mainly occur in cells of different location in GCs, namely either in centroblasts or in superficial centrocytes, respectively. Outside GCs, CD30 and AID (diffuse intracellular staining) are most strongly expressed in two different types of large B lymphoblasts. AID^++^ and CD30^++^ cells primarily occur in the T/B area surrounding the follicles and in the T-cell zones. In this location, AID^++^CD30^−^ cells and double-positive cells were unequivocally identified. An AID^−^CD30^++^ cell type was likely to occur, but the number of double-positive cells could not be investigated in detail. We suppose that strong and uniform expression of AID in CD30^−^ B cells outside GCs indicates the presence of functionally active enzyme related to Ig class switching. The amount of AID present in extrafollicular B lymphoblasts must be much larger than in centroblasts.

The majority of the AID^++^ cells in the T/B area and in the adjacent T-cell zone is positive for CD20, and may thus correspond at least partly to the CD20^+^ “interfollicular large B cells” described in human tonsils by Marafioti et al. (2003). These cells were microdissected and described to contain somatically mutated Ig genes. Weniger et al. (2018) selected CD20^+^CD38^low^ B blasts strongly expressing CD30 from tonsil cell suspensions and classified these cells as post-GC memory cells, which are being re-activated by T cells. It is likely that the cells described by both authors are identical. It has, however, to be kept in mind, that the expression of CD30 in the cells described by Marafioti et al. (2003) was not clear and that the authors found the majority of the CD30^++^ extrafollicular cells to be CD20^−^. This corresponds to our findings. In addition, we suppose that extrafollicular cells most strongly staining for AID or for CD30 represent different populations with only minor overlap, which fits the results of Cattoretti et al. (2006). At the first glance, our staining results seem to be in contrast to the findings of Weniger et al. (2018). The results of all authors studying strong extrafollicular AID and CD30 expression may, however, not be discrepant if the extrafollicular CD30^++^ cells in tonsils represent a cell population slowly downmodulating CD20 when differentiating towards plasmablasts. Jourdan et al. (2011) have shown in vitro that CD30 is expressed by CD20^−^ pre-plasmablasts, before the cells turn into CD38^++^CD138^−^ plasmablasts and further into CD38^++^CD138^++^ plasma cells. Thus, the loss of AID, of CD20, of CD30, and, finally, of proliferative capacity may represent a continuum during plasma cell differentiation in extrafollicular locations.

The CD20^−(or ±)^CD30^++^ stage of B-cell differentiation is absent or at least hardly detectable by immunohistology in GCs, which may represent one of the differences among intra- and extrafollicular PC differentiation. The CD30^++^ extrafollicular cells are often closely located near the dark zone of GCs. It cannot be excluded that these cells derive from the superficial CD30^+^ B-cell blasts found in the dark zone of certain—but not all—GCs. These cells might quickly downmodulate CD20 and further up-regulate CD30, when leaving the GC via the superficial dark zone. Such a scenario would imply combined intra- and extrafollicular differentiation with the help of intra- and extrafollicular T cells. Thus, two local pathways may lead to PC differentiation in tonsils, one pathway occurring entirely in GCs and a second one comprising combined GC plus extra-GC differentiation. The second pathway may only be relevant for a minor and very special B-lymphoblast population. Further information should be gathered on the relationship among CD20^+^CD30^+^ GC and CD20^−(or±)^CD30^++^ extrafollicular B-cell blasts to clarify the question, whether the latter cells are re-activated memory cells (Weniger et al. 2018) directly derived from nearby GCs or from other origins.

Finally, it needs to be mentioned that our study does not exclude a non-T-/non-B-cell derivation of the extrafollicular CD30^++^ cells. However, the phenotypic studies by others and the morphological similarity among intra- and extrafollicular CD30^+/++^ cells strongly argue against such an assumption. In both locations, many of these cells represent large Ki-67^+^ blasts with a morphologically unique and prominent CD30 positivity in the Golgi area. The CD30^+^ cells at the surface of the GC clearly express CD20.

The mantle zone usually does not harbour accumulations of plasma cells or CD30^+^ B blasts in most tonsil follicles. Thus, these cell types obviously do not migrate via the mantle zone. As the surface of the dark zone and of the dark zone/light zone border is almost devoid of a mantle zone, this location is very likely to represent the predominant exit area of GCs both for plasma cells and for CD30^+^ B blasts. We did not only observe apparent bridges of plasma cells across the dark zone border, but CD30^+/++^ B-cell blasts also appeared to be in continuity from the dark zone surface to extrafollicular CD30^++^ cells. Thus, intra-GC and extra-GC CD30^+^ cell types may be related, as also speculated by others (Cattoretti et al. 2006).

**Fig. 11:**
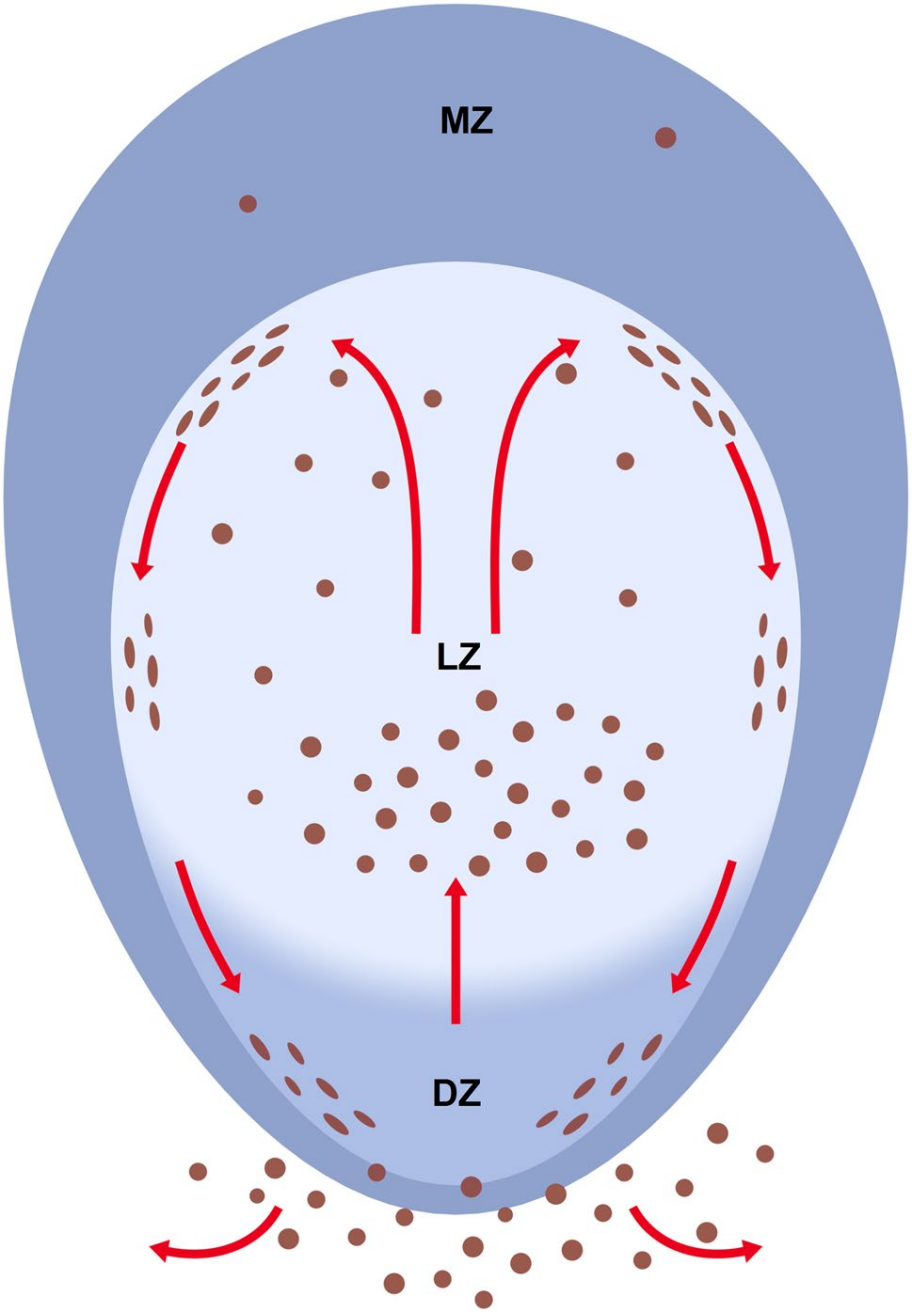
Hypothetical migration of CD38^++^icIg^+^ PCs (brown) in tonsil GCs. *MZ* mantle zone, *LZ* light zone, *DZ* dark zone

On the basis of cellular dynamics as established for acute GC reactions in mice (De Silva et al. 2015, Mesin et al. 2016, Victora et al. 2010) and with the caveat that such dynamics may not adequately reflect chronic B-cell reactions in humans, our results are compatible with the following hypothesis (Fig. 11):

In the process of cyclic re-entry in GCs, centrocytes arising from centroblasts up-regulate CD30 and down-regulate AID when entering the light zone. There is a tendency for the expression of CD30 and AID being mutually exclusive, although we cannot exclude a population of double-positive cells. CD30^+^ B-cell blasts migrate to the light zone surface (i.e., the outer zone), where CD30 is most strongly expressed. Then, these cells proceed back towards the dark zone crawling along the GC surface. It may be that GC *T*_fh_ cells, which are most densely arranged at the surface of the light zone, play a role in induction of CD30. Most cells approaching the dark zone at the surface of the GC again up-regulate AID and downmodulate CD30 at some distance from the centre of the dark zone, which leads to the dish-shaped arrangement of AID^+^ cells seen in some sections. At some moment during cyclic re-entry, centroblasts convert to plasmablasts. In chronically persisting GCs, this cell type is relatively rare.

PCs finally accumulate in the basal light zone, where they are retained for variable time by FDCs with high antigen density and optimal blood supply. Sometimes, PCs form clusters near capillaries and pericapillary FDCs, which may promote plasmablast to PC differentiation. More mature PCs would then diffuse across the apical light zone finally reaching the GC surface. They stick to this surface, but in most cases do not cross it when migrating back towards the surface of the dark zone either to leave the GC or to continue further rounds of superficial migration. This movement guarantees an even distribution of secreted Ig from newly formed plasma cells both deep inside and at the surface of GCs for exchange with pre-existing Ig in immune complexes on FDCs. We hypothesise that the major output of tonsil GCs to the local surroundings consists of PCs and CD30^++^ B lymphoblasts. It can, however, not be excluded that some intra-GC plasma cells become permanently resident cells to maintain a basal level of high-affinity Ig inside the GC. This scenario may not only be valid for tonsils, but also for other human secondary lymphatic organs with persisting GCs.

## References

[CR1] Arpin C, de Bouteiller O, Razanajaona D, Fugier-Vivier I, Brière F, Banchereau J (1998). The normal counterpart of IgD myeloma cells in germinal center displays extensively mutated IgVH gene, Cµ–Cδ switch, and λ light chain expression. J Exp Med.

[CR2] Brachtel EF, Washiyama M, Johnson GD, Tenner-Racz K, Racz P, MacLennan IC (1996). Differences in the germinal centres of palatine tonsils and lymph nodes. Scand J Immunol.

[CR3] Cattoretti G (2013). MYC expression and distribution in normal mature lymphoid cells. J Pathol.

[CR4] Cattoretti G, Büttner M, Shaknovich R, Kremmer E, Alobeid B, Niedobitek G (2006). Nuclear and cytoplasmic AID in extrafollicular and germinal center B cells. Blood.

[CR5] Curran RC, Jones EL (1977). Immunoglobulin-containing cells in human tonsils as demonstrated by immunohistochemistry. Clin Exp Immunol.

[CR6] De Silva NS, Klein U (2015). Dynamics of B cells in germinal centres. Nat Rev Immunol.

[CR7] Fallini B, Pileri S, Pizzolo G, Dürkop H, Flenghi L, Stirpe F (1995). CD30 (Ki-1) molecule: a new cytokine receptor of the tumor necrosis factor receptor superfamily as a tool for diagnosis and immunotherapy. Blood.

[CR8] Fallini B, Fizzotti M, Pucciarini A, Bigerna B, Marafioti T, Gambacorta M (2000). A monoclonal antibody (MUM1p) detects expression of the MUM1/IRF4 protein in a subset of germinal center B cells, plasma cells, and activated T cells. Blood.

[CR9] Garimilla S, Nguyen DC, Halliley JL, Tipton C, Rosenberg AF, Fucile CF (2019). Differential transcriptome and development of human peripheral plasma cell subsets. JCI Insight.

[CR10] Greiner A, Tobollik S, Buettner M, Jungnickel B, Herrmann K, Kremmer E, Niedobitek G (2005). Differential expression of activation-induced cytidine deaminase (AID) in nodular lymphocyte-predominant and classical Hodgkin lymphoma. J Pathol.

[CR11] Gutzeit C, Chen K, Cerutti A (2018). The enigmatic function of IgD: some answers at last. Eur J Immunol.

[CR12] Habermann AM, Gonzalez DG, Wong P, Zhang TT, Kerfoot SM (2019). Germinal center B cell initiation, GC maturation, and the coevolution of its stromal cell niches. Immunol Rev.

[CR13] Hardie DL, Johnson GD, Khan M, MacLennan IC (1993). Quantitative analysis of molecules which distinguish functional compartments within germinal centers. Eur J Immunol.

[CR14] Ise W, Fuji K, Shiroguchi K, Ito A, Kometani K, Takeda K (2018). T follicular helper cell-germinal center B cell interaction strength regulates entry into plasma cell or recycling germinal center cell fate. Immunity.

[CR15] Jourdan M, Caraux A, Caron G, Robert N, Fiol G, Rème T (2011). Characterization of a transitional preplasmablast population in the process of human B cell to plasma cell differentiation. J Immunol.

[CR16] Khodadadi L, Cheng Q, Radbruch A, Hiepe F (2019). The maintenance of memory plasma cells. Front Immunol.

[CR17] Klein U, Casola S, Cattoretti G, Shen Q, Lia M, Ludwig T (2006). Transcription factor IRF4 controls plasma cell differentiation and class-switch recombination. Nat Immunol.

[CR18] Korsrud FR, Brandtzaeg P (1980). Immune systems of human nasopharyngeal and palatine tonsils: histomorphometry of lymphoid components and quantification of immunoglobulin-producing cells in health and disease. Clin Exp Immunol.

[CR19] Kräutler NJ, Suan D, Butt D (2017). Differentiation of germinal center B cells into plasma cells is initiated by high-affinity antigen and completed by Tfh cells. J Exp Med.

[CR20] Küppers R, Zhao M, Hansmann ML, Rajewsky K (1993). Tracing B cell development in human germinal centers by moleculer analysis of single cells picked from histological sections. EMBO J.

[CR21] Lennert K, Caesar R, Müller-Hermelink HK, Cottier H, Odartchenko N, Schindler R, Congdon CC (1967). Electron microscopic studies of germinal centres in man. Germinal centers in immune responses.

[CR22] Liu Y-J, de Bouteiller O, Arpin C, Brière F, Galibert L, Ho S (1996). Normal human IgD^+^IgM^+^ germinal center B cells can express up to 80 mutations in the variable region of their IgD transcripts. Immunity.

[CR23] Maeda K, Matsuda M, Suzuki H, Saitoh HA (2002). Immunohistochemical recognition of human follicular dendritic cells (FDCs)in routinely processed paraffin sections. J Histochem Cytochem.

[CR24] Marafioti T, Jones M, Facchetti F, Diss TC, Du MQ, Isaacson PG (2003). Phenotype and genotype of interfollicular large B cells, a subpopulation of lymphocytes often with dendritic morphology. Blood.

[CR25] Mei HE, Yoshida T, Muehlinghaus G, Hiepe F, Dörner T, Radbruch A, Hoyer BF (2007). Phenotypic analysis of B-cells and plasma cells. Methods Mol Med.

[CR26] Mesin L, Ersching J, Victora GD (2016). Germinal center B cell dynamics. Immunity.

[CR27] Methor SP, Litzler LC, Trajtenberg F, Zahn A, Robert F, Pelletier J (2015). Consecutive interactions with HSP90 and eEF1A underlie a functional maturation and storage pathway of AID in the cytoplasm. J Exp Med.

[CR49] Mohr E, Serre K, Manz RA, Cunningham AF, Khan M, Hardie DL, Bird R, MacLennan ICM (2009). Dendritic cells and monocyte/macrophages that create the IL-6/APRIL-rich lymph node microenvironments where plasmablasts mature. J Immunol.

[CR28] Nieuwenhuis P, Opstelten D (1984). Functional anatomy of germinal centers. Am J Anat.

[CR29] Odendahl M, Mei H, Hoyer BF, Jacobi AM, Hansen A, Muehlinghaus G (2005). Generation of migratory antigen-specific plasma blasts and mobilization of resident plasma cells in a secondary immune response. Blood.

[CR30] Patenaude A-M, Orthwein A, Hu Y, Campo VA, Kavli B, Buschiazzo A, Noia JM (2009). Active nuclear import and cytoplasmic retention of activation-induced deaminase. Nat Struct Mol Biol.

[CR31] Pezzati P, Stanisz AM, Marshall JS, Bienenstock J, Stead RH (1992). Expression of nerve growth factor receptor immunoreactivity on follicular dendritic cells from human mucosa associated lymphoid tissues. Immunology.

[CR32] Pitcher CJ, Hagen SI, Walker JM, Lum R, Mitchell BL, Maino VC (2002). Development and homeostasis of T cell memory in rhesus macaque. J Immunol.

[CR33] Randall TD (2010). Bronchus-associated lymphoid tissue (BALT) structure and function. Adv Immunol.

[CR34] Schwarting R, Gerdes J, Dürkop H, Falini B, Pileri S, Stein H (1989). BER-H2: a new anti-Ki-1 (CD30) monoclonal antibody directed at a formol-resistant epitope. Blood.

[CR35] Seifert M, Steimle-Grauer SA, Goossens T, Hansmann M-L, Bräuninger A, Küppers R (2009). A model for the development of human IgD-only B cells: Genotypic analyses suggest their generation in superantigen driven immune reponses. Mol Immunol.

[CR36] Smith HC (2017). RNA binding to APOBEC deaminases; Not simply a substrate for C to U editing. RNA Biol.

[CR37] Steiniger B, Timphus EM, Jacob R, Barth PJ (2005). CD27^+^ B cells in human lymphatic organs: re-evaluating the splenic marginal zone. Immunology.

[CR38] Steiniger B, Timphus EM, Barth PJ (2006). The splenic marginal zone in humans and rodents—an enigmatic compartment and its inhabitants. Histochem Cell Biol.

[CR39] Steiniger B, Trabandt M, Barth PJ (2011). The follicular dendritic cell network in secondary follicles of human palatine tonsils and spleens. Histochem Cell Biol.

[CR40] Steiniger BS, Seiler A, Lampp K, Wilhelmi V, Stachniss V (2014). B lymphocyte compartments in the human splenic red pulp: capillary sheaths and periarteriolar regions. Histochem Cell Biol.

[CR41] Steiniger BS, Wilhelmi V, Seiler A, Lampp K, Stachniss V (2014). Heterogeneity of stromal cells in the human splenic white pulp. Fibroblastic reticulum cells, follicular dendritic cells and a third superficial stromal cell type. Immunology.

[CR42] Steiniger BS, Wilhelmi V, Berthold M, Guthe M, Lobachev O (2018). Locating human splenic capillary sheaths in virtual reality. Sci Rep.

[CR43] Thompson SJ, Schattemann GC, Gown AM, Bothwell M (1989). A monoclonal antibody against nerve growth factor receptor. Immunohistochemical analysis of normal and neoplastic human tissue. Am J Clin Pathol.

[CR44] Victora GD, Schwickert TA, Fooksman DR, Kamphorst AO, Meyer-Hermann M, Dustin ML, Nussenzweig MC (2010). Germinal center dynamics revealed by multiphoton microscopy with a photoactivatable fluorescent reporter. Cell.

[CR45] Weniger MA, Tiacci R, Schneider S, Arnolds J, Rüschenbaum S, Duppach J (2018). Human CD30^+^ B cells represent a unique subset related to Hodgkin lymphoma cells. J Clin Invest.

[CR46] Zhang Y, Meyer-Hermann M, George LA (2013). Germinal center B cells govern their own fate via antibody feedback. J Exp Med.

[CR47] Zhang Y, Garcia-Ibanez L, Toellner KM (2016). Regulation of germinal center B-cell differentiation. Immunol Rev.

[CR48] Zhang Y, Tech L, George LA, Acs A, Durrett RE, Hess H (2018). Plasma cell output from germinal centers is regulated by signals from Tfh and stromal cells. J Exp Med.

